# Integrating Kolmogorov-Arnold networks and sparse attention for robust visual plant disease symptom identification across diverse agricultural crops

**DOI:** 10.3389/fpls.2026.1855159

**Published:** 2026-07-02

**Authors:** Yunqin Shen, Mengyuan Zhu, Tao Hu, Peng Xu, Yutao Zhu, Longguo Wu, Laixiang Xu, Rajveer Dhillon

**Affiliations:** 1 School of Computer and Artificial Intelligence, Henan University of Urban Construction, Pingdingshan, China; 2 School of Computer and Artificial Intelligence and School of Life Sciences and Engineering, Pingdingshan Academy of Agricultural Sciences, Henan University of Urban Construction, Pingdingshan, China; 3 School of Spatiotemporal Information Engineering, Henan University of Urban Construction, Nanchang, China; 4 Key Laboratory of Modern Agricultural Equipment of Jiangxi Province, Jiangxi Agricultural University, Nanchang, China; 5 School of Life Sciences and Engineering, Henan University of Urban Construction, Pingdingshan, China; 6 Faculty of Agriculture, Forestry and Ecology, Ningxia University, Yinchuan, China; 7 Agricultural Research and Development Program, Central State University, Wilberforce, OH, United States

**Keywords:** computer-vision, deep learning, dynamic sparse attention mechanism, improved vision transformer, visual symptom identification

## Abstract

**Introduction:**

Accurate and rapid diagnosis of plant leaf disease symptoms is critical for sustainable agricultural crop production, yet traditional methods often lack efficiency and robustness under field conditions.

**Methods:**

Here, we propose a deep learning framework based on an improved Vision Transformer architecture that integrates a dynamic sparse attention mechanism, termed KBTNet, for targeted feature extraction in symptom-affected regions of leaf images. The model incorporates a learnable nonlinear enhancement module to capture subtle visual disease symptom variations such as lesions, discoloration patterns, and spot distributions, and a lightweight Transformer design to reduce computational cost.

**Results:**

Evaluated on a multisource dataset containing soybean and tomato leaf images representing diverse disease symptom patterns, our approach achieved 93.19% classification accuracy, outperforming current state-of-the-art models. Additional evaluations on public plant disease datasets from multiple crops further demonstrate the model's ability to recognize disease symptom patterns across diverse crop species.

**Discussion:**

The proposed framework achieves stable performance across diverse crop and disease symptom categories, maintains high efficiency under reduced parameter complexity, and exhibits strong potential for realtime field diagnostics on edge devices. This work provides a scalable and efficient tool for plant disease symptom detection and classification and supports the integration of visionbased intelligence into crop disease monitoring and management systems.

## 1 Introduction

Amid rapid global urbanization and population growth, food security has emerged as a critical global challenge. Agricultural crops serve as essential sources of edible oil and plant protein (Liu et al., 2025), forming a vital component of global agricultural systems due to their high yield and quality requirements (Su et al., 2024). However, these crops are highly susceptible to various leaf diseases during growth, including viral infections, leaf spot, and bacterial wilt. These threats can severely compromise both yield and crop quality, leading to considerable economic losses. Consequently, developing efficient and accurate methods for detecting and classifying crop leaf diseases and their visual symptoms is imperative for enhancing agricultural productivity, ensuring food security, and promoting sustainable agricultural development.

Plant leaf diseases are typically expressed through characteristic visual symptoms on leaves, including lesions, spots, chlorosis, mosaic patterns, and tissue necrosis (Pfordt and Paulus, 2025). These visible symptom patterns are commonly caused by fungal, bacterial, or viral pathogens that disrupt plant physiological processes such as chlorophyll synthesis, cellular integrity, and photosynthesis. For example, fungal infections often produce necrotic lesions or spots on leaves, bacterial infections may induce blights and chlorotic halos, while viral infections frequently generate mosaic or mottled patterns accompanied by leaf deformation and growth suppression (Jemo et al., 2023). Accurate recognition of these visual symptoms provides an important basis for disease detection and management in crop production systems.

Conventional identification of plant leaf diseases often relies on handcrafted feature extraction combined with computer vision techniques. This approach typically involves extracting discriminative features such as shape, color, and texture from leaf images using predefined algorithms. For instance, color histograms capture the overall color distribution, while gray-level co-occurrence matrices (GLCMs) characterize textural properties, helping distinguish diseased from healthy leaf regions. These features are subsequently classified using traditional machine learning (Mansoor et al., 2025) models like support vector machines (SVM) and k-nearest neighbors (KNN) to achieve disease recognition. Previous studies have reported promising results: Kumar et al. (2025) applied a predator search algorithm with weighted random logistic Bayesian support vectors, attaining 98.4% recognition accuracy; Rivera-Romero et al. (2025) developed a non-invasive remote sensing technique for detecting fungal infections in soybeans, reporting 98% accuracy, and Leal-Lara et al. (2025) integrated fuzzy logic with neural networks to identify 15 types of crop leaf diseases, achieving 93.81% accuracy.

However, such manually engineered features depend heavily on expert knowledge, and their effectiveness varies across disease types. Color histograms and GLCM, for instance, often fail to capture subtle, complex disease symptoms, especially at early stages like initial soybean rust infection, where visual cues are minimal (Feng et al., 2024). This limitation can lead to inadequate feature representation and impaired classification performance. Furthermore, traditional classifiers are sensitive to training data distribution and prone to overfitting on unrepresentative samples, which undermines generalizability (Alves et al., 2025).

Recent advances in artificial intelligence have established deep learning (Gan et al., 2025) as a mainstream approach for plant disease recognition based on visual symptom patterns in leaf images. By leveraging deep neural networks trained on large-scale datasets, these methods automatically learn discriminative features, enhancing both accuracy and robustness. Vision-based architectures, particularly Vision Transformers and convolutional neural networks, enable automated detection and classification of plant diseases by identifying characteristic visual symptoms such as lesions, discoloration, and morphological changes. For instance, Chen et al. (2025a) developed an improved YOLOv8 model achieving 96.9% mAP50 in soybean disease identification. Song et al. (2025) introduced a cellular P system with membrane rules attaining 98.43% accuracy. Bhujade et al. (2025) proposed a capsule network with positional attention mechanisms reporting 98% accuracy, while Hang et al. (2024) applied a refined lightweight network achieving 94.27%. Huang et al. (2024) designed a hypergraph-based membrane computing network that also reached 98% accuracy. Despite these advances, further improvements in recognition performance and efficiency are needed for practical agricultural deployment.

Motivated by these observations, we propose a plant leaf disease recognition method based on an improved Vision Transformer (ViT) architecture. The proposed model integrates a Kolmogorov-Arnold Network (KAN) for enhanced nonlinear representation and a dynamic sparse attention mechanism inspired by BiFormer. Although Vision Transformer has demonstrated strong performance across various computer vision tasks, its high computational requirements hinder practical deployment in agricultural field environments. To address this limitation, we introduce a deeply optimized ViT variant that incorporates KAN to strengthen multi-scale feature representation and employs BiFormer’s dynamic sparse attention for efficient extraction of disease-related visual symptom features, thereby reducing computational overhead. The main contributions of this work are summarized as follows:

1. The classical ViT is optimized to reduce computational complexity while improving inference speed and efficiency, enabling deployment on resource-constrained edge devices.
2. Integration of the BiFormer module allows the model to dynamically focus on key symptomatic regions associated with disease in leaf images, minimizing redundant computation and enhancing recognition performance under complex backgrounds.
3. Incorporation of KAN strengthens the model’s multi-scale feature representation capability and improves robustness to challenging conditions, including leaf deformation and occlusion.

The proposed model adopts a three-stage architecture comprising a lightweight Vision Transformer (ViT) for foundational feature extraction, a dynamic sparse attention mechanism for regional emphasis, and adaptive nonlinear transformation via the Kolmogorov-Arnold Network (KAN). A hierarchical interaction mechanism incorporating skip connections facilitates the complementary fusion of sparse local features with global semantic information. This design mitigates overfitting on small crop disease datasets and enhances feature discriminability through modular synergy, achieving an optimal balance between accuracy and efficiency.

In terms of the classification mechanism, the model integrates sparse local perception, global context modeling, and adaptive nonlinear enhancement to address specific challenges in plant disease recognition. It effectively captures complex multi-scale visual crop disease symptom patterns, accentuates discriminative features such as lesion edges and color variations, and reliably discriminates between healthy tissue and multiple disease categories. Compared to conventional approaches, the proposed method demonstrates superior adaptability and discriminative capacity when handling complex and variable image characteristics encountered in agricultural production systems.

Although the initial focus of this study is soybean leaf diseases on the field data collected by our team, the experimental framework also incorporates public datasets from tomato disease images and additional diverse crops that show similar visual leaf symptoms to evaluate cross-crop generalization capability. By testing the model on multiple plant species exhibiting diverse visual symptom patterns, the study assesses the robustness and adaptability of the proposed architecture for recognizing disease symptoms across diverse crops. In summary, this study introduces a lightweight and efficient network for plant leaf disease recognition with a focus on horticultural applications. Through multi-dimensional structural optimization, the model achieves a synergistic balance between accuracy and computational efficiency. It accurately identifies diseased regions and classifies multiple disease types while maintaining low parameter complexity and high computational efficiency, enabling deployment on edge devices such as portable detectors and agricultural drones. We provide a practical and scalable solution for real-time horticultural crop disease surveillance under field conditions.

## 2 Literature reviews

In precision agriculture, deep learning-based visual symptom recognition for crop leaf diseases is integral to smart systems such as see and spray technologies (Shafay et al., 2025). By detecting patterns such as lesions, spots, chlorosis, necrosis, and mosaic discoloration in leaf imagery, these systems enable real-time crop health monitoring and support advanced crop protection strategies, thereby enhancing field management efficiency, disease control, yield prediction, and food security. Current disease identification methods fall into traditional machine learning and deep learning categories. Accordingly, we organize mainstream approaches into three groups: traditional machine learning models, deep learning architectures (Fan et al., 2025), and attention-based mechanisms. **Table 1** systematically compares their advantages and limitations in agricultural contexts.

**Table 1:** Comparison of different methods.

Technical category	Methods	Advantages	Limitations	Relevance to this study
Machine learning	Logistic Regression/Decision Trees	High transparency and fast training on limited data	Dependent on feature engineering	Baseline to evaluate automated feature extraction benefits
Basic CNN	ResNet18/AlexNet	Reliable performance and easy fine-tuning	Computationally intensive for edge deployment	Standard for comparing efficiency gains
Lightweight CNN	ShuffleNetV2/SqueezeNet	Good balance of accuracy and speed	Struggles with complex or multi-scale objects	Test hybrid attention-convolution efficiency
Attention model	ViT-Tiny/EfficientFormer	Captures long-range dependencies effectively	High memory use; not optimized for field conditions	Inspiration for lightweight attention mechanisms

Traditional methods for plant leaf disease identification typically involve image preprocessing, handcrafted feature extraction, and classification. For instance, Ferraz et al. (2024) applied random forests to classify soybean plants at the R7 stage, achieving 94% accuracy. Mumtaz et al. (2025) implemented a Gaussian distribution-based classifier with 92% recognition accuracy, while Zhao et al. (2025) adopted hyperspectral imaging to distinguish healthy from infected samples, attaining 95.39% accuracy. Wei et al. (2025) used SVM to classify mold severity levels, reaching 98.27% accuracy. Elbasi et al. (2024) enhanced feature extraction and selection for leaf image classification on the Kew Leaf dataset, achieving 89.48% accuracy. Alsakar et al. (2024) integrated color texture with local binary patterns and color correlation maps, reporting over 99% accuracy in rice image classification.

Although these traditional methods perform well in controlled agricultural environments, they exhibit limited generalization capability and often fail to handle real-world image noise and intra-class variations effectively. Performance declines significantly under challenging field conditions common in agricultural environments, such as varying illumination, partial occlusion, or physical leaf damage. Moreover, due to their reliance on handcrafted features, these methods generally underperform in the high-noise environments and complex backgrounds typical of open-field agricultural production.

Deep learning-based plant disease recognition via visual symptom analysis has become a mainstream technology in modern agriculture, offering substantial advantages in feature extraction and processing efficiency. By autonomously learning complex patterns from data, deep learning (Li et al., 2025a) achieves notable improvements in classification accuracy and robustness under challenging field conditions, such as leaf occlusion, morphological variation, and low illumination. These models learn discriminative representations of visual symptoms directly from leaf images, including lesion shapes, discoloration patterns, and spatial texture variations. Numerous studies have demonstrated the effectiveness of deep learning in this domain. Liu et al. (2024) classified diseased soybean leaves with 98.7% accuracy. Pan et al. (2023) proposed a two-stage feature aggregation network achieving 98.18% accuracy. Novel architectures have further advanced performance: Baek (2025) introduced a multi-visual Transformer model based on attention mechanisms, achieving 99% accuracy; Chen and Liu (2025b) presented CBSNet for potato disease recognition with 92.04% mean accuracy; and Karthik et al. (2025) designed a hybrid Transformer-CNN model attaining 97.66% accuracy. Other notable contributions include those by Yadav and Tewari (2025); Kavitha and Chinnaiah (2025); Bais et al. (2024); Nobel et al. (2024), and Yousafzai et al. (2025), with reported accuracy ranging from 90% to 99.65% across diverse crops and public datasets, including PlantVillage.

Transformer-based vision architectures have recently gained increasing attention in plant disease symptom recognition because their self-attention mechanisms capture long-range spatial relationships among visual features such as lesion distributions and discoloration patterns across the leaf surface (Mehdipour et al., 2026). Despite remarkable progress in plant disease identification, where accuracy rates frequently exceed 95% and even reach 99% in complex scenarios, deep learning (Zhang et al., 2021) still faces several challenges for agricultural adoption. These include high dependency on labeled data, limited adaptability to real-world environmental variations in agricultural systems, difficulties in achieving real-time inference without sacrificing accuracy, and constrained cross-regional generalization.

In conclusion, intelligent recognition of crop leaf diseases based on visual symptom patterns has evolved from traditional manual-feature-based approaches to deep learning-driven systems. While advanced architecture has substantially improved accuracy and complex-scene adaptability for agricultural applications, current methods still face critical challenges, including high data dependency, limited robustness under real-world field conditions, and difficulties in balancing real-time inference with recognition performance. Furthermore, many existing approaches have been developed for single crop species, while practical agricultural monitoring systems must recognize disease symptoms across diverse crops and varying field environments (Li et al., 2025b). Future efforts should prioritize developing lightweight, highly generalizable models capable of operating under practical field conditions to advance the deployment and applicability of crop disease symptom recognition technologies across diverse sets of crops.

## 3 Materials and methods

### 3.1 Improving vision transformer

**Figure 1:**
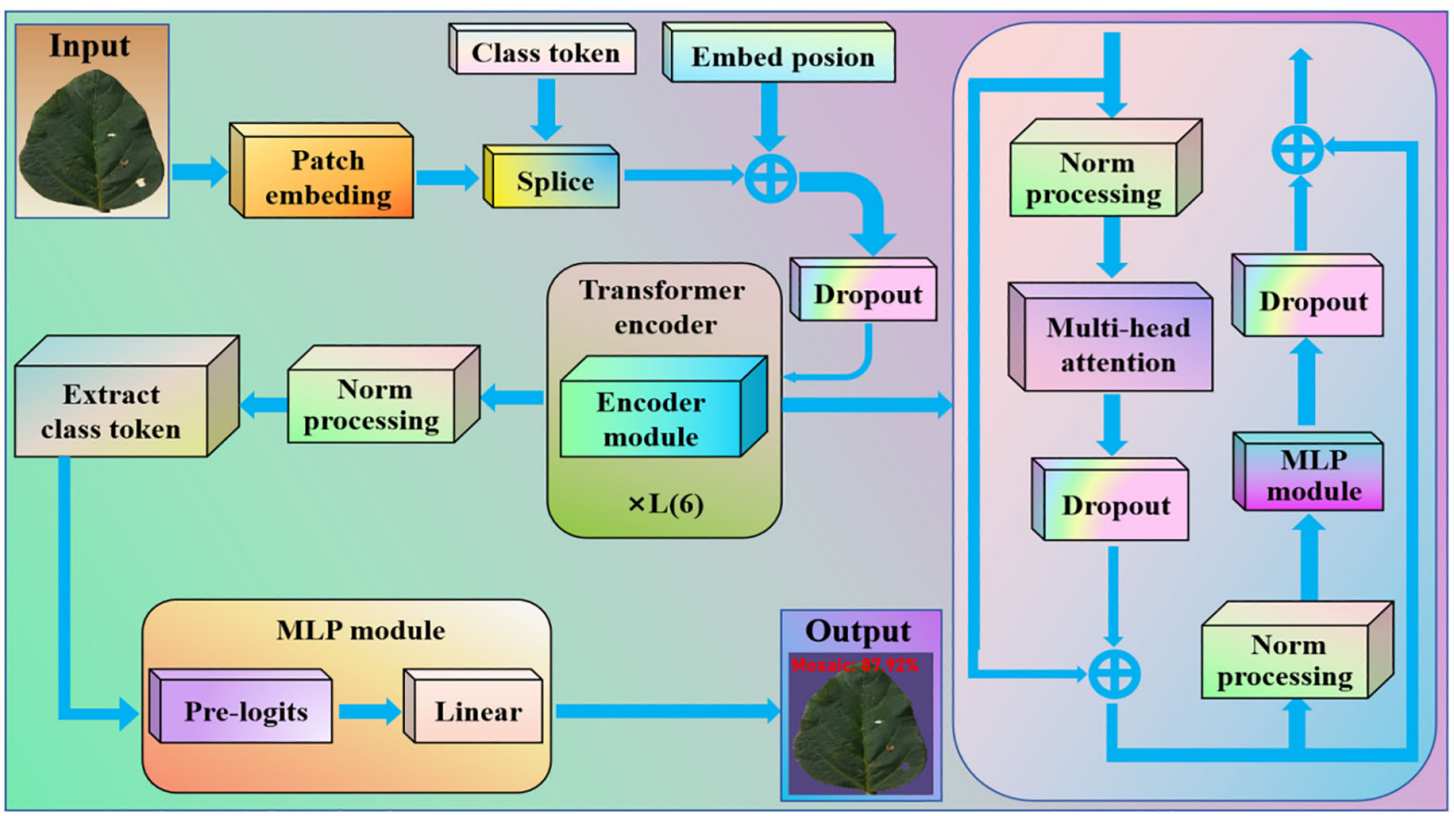
illustrates the improved vision transformer architecture. The input image undergoes patch embedding with positional encoding, followed by a prepended class token. The transformer encoder, comprising multi-head self-attention and normalization layers, performs feature extraction of visual symptom features from leaf images. The final output provides classification of disease symptom categories with confidence scores.

Leveraging a Vision Transformer as the backbone, this study aims to recognize soybean leaf disease symptoms by capturing long-range dependencies and global context through self-attention, which can detect the same visual symptoms in other crops as well. However, the standard ViT is limited by high computational complexity and parameter volume, increasing overfitting risks on limited datasets and hindering real-time use. To address this while preserving ViT’s strengths, we introduce targeted optimizations: reducing the initial embedding dimension to minimize redundancy and sharpen focus on visually observable symptom regions such as lesions, spots, and discoloration patterns, and decreasing the number of Transformer layers and attention heads to compress model depth and width, thus mitigating overfitting to background features. These modifications lower training and inference costs, improve generalization across environments and growth stages, and achieve a better efficiency-accuracy balance. The refined architecture is shown in **Figure 1**.

Within the Transformer encoder, each layer processes the input feature sequence through a self-attention mechanism followed by an MLP block. The output is normalized and combined with the original input via residual connections, establishing the following mapping between input and output features.

The input image is divided into N patches, which are linearly mapped into d-dimensional vectors and augmented with positional encodings. The process can be defined in Equation 1:

(1) $$z_{0}=[X_{class};X_{p}^{1}E;X_{p}^{2}E;\ldots ;X_{p}^{N}E]+E_{pos}$$

where $\text{E}\in {\text{R}}^{({\text{P}}^{2}\cdot \text{C})\times \text{d}}$ is the projection matrix that transforms image patches into feature embeddings, and ${\text{E}}_{\text{pos}}\in {\text{R}}^{(\text{N}+1)\times \text{d}}$ is the positional encoding that represents the spatial position of each patch within the leaf image.

The input feature sequence ${\text{X}}^{\text{l}-1}\in {\text{R}}^{\text{N}\times \text{d}}$, where N denotes the number of patches plus one class token, and d represents the embedding dimension, is linearly projected to generate the Query (Q), Key (K), and Value (V) matrices. The Q, K, and V can be computed in Equation 2:

(2) $$\left\{ \begin{array}{l} Q=X^{l-1}W_{Q},W_{Q}\in R^{d\times d_{k}}\\ K=X^{l-1}W_{K},W_{K}\in R^{d\times d_{k}}\\ V=X^{l-1}W_{V},W_{V}\in R^{d\times d_{k}} \end{array}\right.$$

where *l* is the layer index, $W_{Q}/W_{K}/W_{V}$, the learnable weight matrix and $d_{k}=d_{v}=d/h$, and *h* means the number of attention heads.

The attention output can be calculated in Equation 3:

(3) $$Attention(Q,K,V)=soft\max(\frac{QK^{T}}{\sqrt{d_{k}}})V$$

After being normalized by LayerNorm, the self-attention output is input into the MLP block for nonlinear transformation. It can be expressed in Equation 4:

(4) $$X_{MLP}^{l}=GELU\;(X_{attn}^{l}W_{1}+b_{1})W_{2}+b_{2}$$

where $W_{1}\in R^{d\times 4d},W_{2}\in R^{4d\times d}$ is the weights of the fully connected layer with an expansion ratio of 4.

The original information is retained in each layer output through residual connections. They can be formulated in Equation 5:

(5) $$X^{l+1}=LayerNorm(X^{l}+DropPath(MLP(X^{l})))$$

where DropPath is stochastic depth regularization, which involves dropping out sub-layer outputs with probability p during training.

Take the feature vector corresponding to the [CLS] token, and output the class probability through a single-layer linear layer. It can be defined in Equation 6:

(6) $$y=soft\max(LayerNorm(X_{0}^{L})W_{head})$$

where $X_{0}^{L}$ is the [CLS] token of the last layer.

Through multi-layer feature transformation, the Transformer encoder extracts high-level semantic representations from images, thus providing discriminative features for downstream tasks, including classification and detection of plant disease symptom patterns. This architecture preserves critical input information while employing self-attention to effectively capture long-range dependencies and global context.

### 3.2 Designing KAN

Although reducing Transformer layers improves efficiency, the standard Vision Transformer’s feed-forward network (FFN) relies on fixed activation functions, limiting its adaptability to subtle nonlinear variations in foliar disease symptoms. To address this, we replace the conventional multilayer perceptron in the FFN with Kolmogorov-Arnold Networks (KAN), which employ learnable univariate functions and spline-based parameterization for more flexible feature transformation. Rooted in the Kolmogorov-Arnold representation theorem, KAN enhances modeling of complex symptom characteristics, including lesion texture and color transitions, while maintaining low computational cost. This design strengthens approximation capacity, making it well suited for fine-grained plant disease recognition.

The primary objective of the KAN architecture is to approximate the input–output mapping in supervised learning tasks. Given a dataset {*x_i_*, *y_i_*}, the goal is to find a function *f*(*x*) that satisfies the following relationship. It can be defined in Equation 7:

(7) $$y_{i}=f(x_{i})$$

The core of KAN lies in finding a suitable data representation, utilizing nested combinations of univariate functions. It can be expressed in Equation 8:

(8) $$f(x)=\sum_{k}\varphi_{k}\left(\sum_{j}w_{jk}x_{j}\right)$$

where *x_j_* is the j-th input feature, *w_jk_* denotes the weight parameter connecting the input *x_j_* and the activation function *ϕ_k_*, and *ϕ_k_* is a nonlinear activation function constructed using B-splines.

In the *l*-th layer of the KAN network, the mapping relationship can be expressed in Equation 9:

(9) $$h^{\left(l\right)}(x)=S^{\left(l\right)}\cdot x+b^{\left(l\right)}$$

where $h^{\left(l\right)}(x)$ is the output of the *l*-th layer, $S^{\left(l\right)}$ denotes the B-spline kernel matrix, which is used to adjust the nonlinear changes of input features representing visual disease symptoms, and $b^{\left(l\right)}$ stands for the bias term.

KAN employs a residual activation function to connect the base kernel function with the B-spline function. It can be described in Equation 10:

(10) φk(x)=∑mαmBm(x)+Res(x)

where *B_m_*(*x*) is the *m*-th B-spline basis function, *α_m_* denotes the weight parameter of the B-spline function, Re *s*(*x*) stands for the residual term, which connects the base kernel function.

and the B-spline function.

### 3.3 Creating BiFormer

While the KAN module enhances nonlinear approximation, it insufficiently mitigates deep feature redundancy and the integration of local details with global context. To address this, we introduce BiFormer dynamic sparse attention, which employs region-aware sparsity to focus computation on salient regions while suppressing irrelevant background. This improves multi-scale feature extraction for plant disease symptoms by constructing query-adaptive sparse patterns, maintaining efficient long-range dependency modeling. This design compensates for KAN’s limitations in global-local coordination, enhancing discriminative capacity for complex disease features.

**Figure 2:**
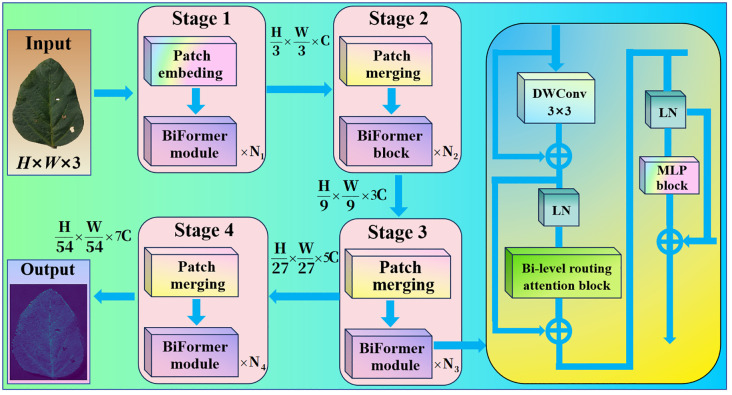
illustrates the BiFormer architecture. Input images are transformed into patch embeddings and processed through four hierarchical stages. Each stage incorporates BiFormer blocks with bi-level routing attention and depthwise convolutions, progressively reducing spatial resolution while enhancing feature representation. The final output yields a H×W×54 feature map.

The architecture and data flow of the BiFormer are described in **Figure 2**.

Compared to alternative sparse attention mechanisms such as Swin Transformer with fixed window attention and CrossFormer with cross-scale attention, the bi-level routing mechanism of BiFormer is more suitable for extracting features of plant disease symptoms. These symptoms are typically sparsely distributed (e.g., scattered lesions) and vary in scale from small spots to large necrotic areas. Moreover, BiFormer offers lower computational complexity, which aligns well with the lightweight design objectives of this study and the requirements for subsequent edge deployment.

This mechanism filters out irrelevant key-value pairs at the coarse-grained region level and It performs attention computation on the filtered regions at the fine-grained token level, thereby effectively reducing computational complexity and enhancing the scalability of the model.

Given a two-dimensional input feature map $X\in R^{H\times W\times C}$, it is first divided into S×S non-overlapping regions, with each region containing $\frac{\text{HW}}{{\text{S}}^{2}}$ feature vectors. This step is achieved by reshaping $X^{r}\in R^{S^{2}\times \frac{HW}{S^{2}}\times C}$ through X.

Then, the query Q, key K, and value V are obtained through linear projection. They can be expressed in Equation 11:

(11) $$Q=X^{r}W^{q},K=X^{r}W^{k},V=X^{r}W^{v}$$

where $W^{q},W^{k},W^{v}\in R^{C\times C}$ are the projection weights of Q, K, and V respectively.

Construct a directed graph to determine the other regions that each region should pay attention to. Firstly, calculate the region-level query Q^r^ and key K^r^ by averaging the query and key for each region. Then, calculate the adjacency matrix *A^r^* by matrix multiplication of Q^r^ and K^r^. It can be computed in Equation 12:

(12) $$A^{r}=Q^{r}{\left(K^{r}\right)}^{T}$$

The elements in the adjacency matrix A^r^ represent the semantic correlation between two regions. Then, the graph is pruned by retaining the top k connections for each region, and the index matrix ${\text{Ir}}_{\in }{\text{N}}^{{\text{S}}^{2}\times \text{k}}$ is obtained using row-wise top k operation. It can be defined in Equation 13:

(13) $${\text{Ir}}^{=}\text{ topkIndex}({\text{Ar}}^{)}$$

The *i*-th row of the index matrix I^r^ contains the indices of the k most relevant regions to the.

*i*-th region.

We utilize a region-to-region routing index matrix I^r^ to implement fine-grained token-to-token Attention. For each region i, we gather the keys and values associated with that region. It can be formulated in Equation 14:

(14) $$K^{g}=gather(K,I^{r}),V^{g}=gather(V,I^{r})$$

Then, we apply an attention mechanism to the collected key-value pairs. It can be defined in Equation 15:

(15) $$O=Attention(Q,K^{g},V^{g})+LCE(V)$$

Among them, LCE(V) is a local context enhancement term achieved through deep convolution.

**Figure 3:**
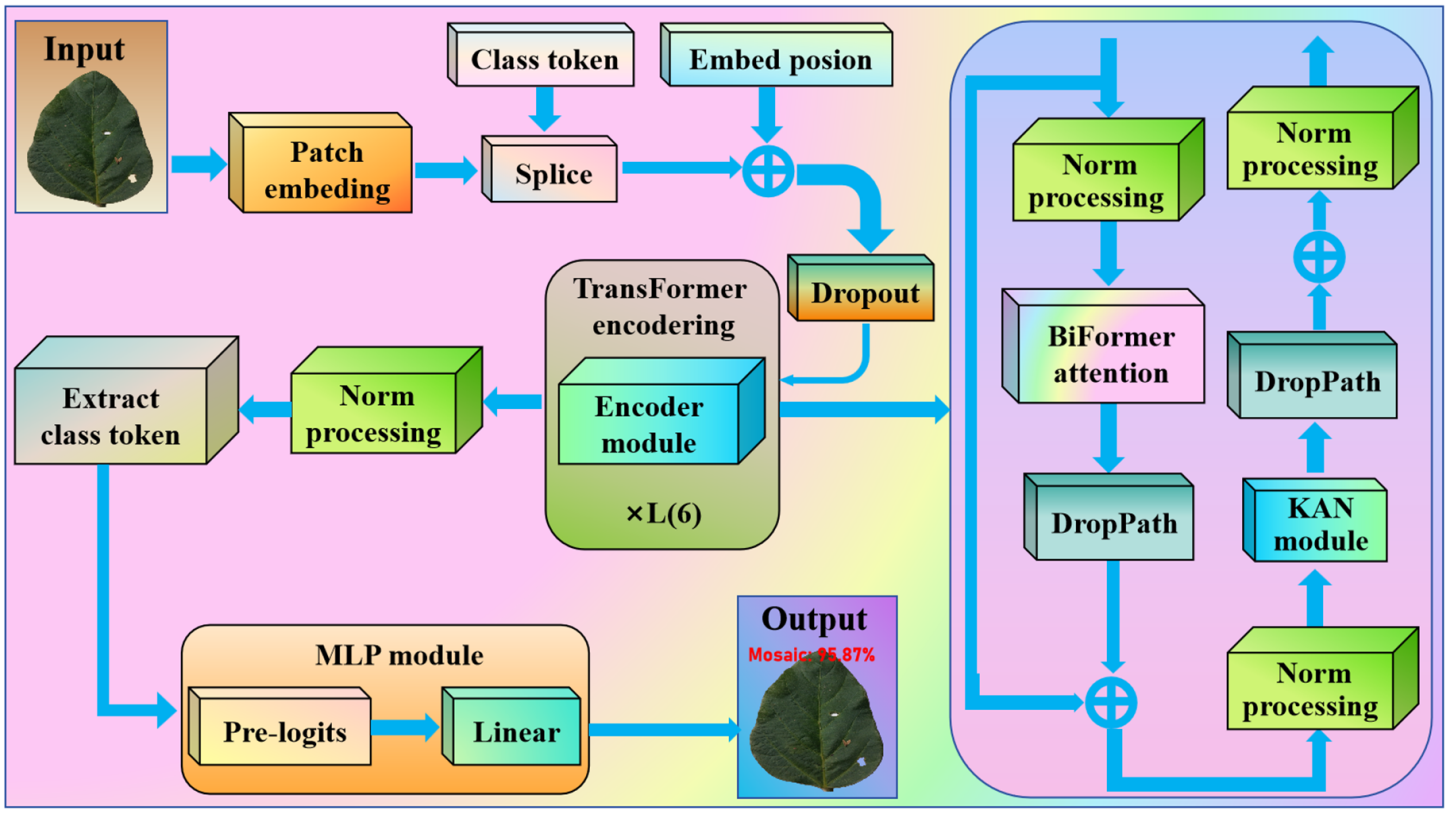
presents the overall soybean leaf disease classification framework. Based on an improved Vision Transformer, it replaces MLP with KAN and incorporates BiFormer attention. The model processes input leaf images and outputs predicted plant disease symptom categories.

Based on the aforementioned analysis, the overall structure of the soybean disease leaf recognition framework designed in this paper is illustrated in **Figure 3**.

The proposed framework first streamlines the Vision Transformer architecture to enhance inference efficiency. It then incorporates Bi-Former attention across Transformer layers to achieve dynamic sparse computation, preserving local focus on symptom regions such as lesions in shallow layers, while modeling global leaf health relationships at deeper levels. Finally, the KAN module replaces standard MLP activations with learnable nonlinear functions, enabling depth-adaptive feature transformation that enhances sensitivity to subtle color and texture variations. This integrated design maintains efficient feature extraction while accelerating convergence and improving generalization capability and robustness.

### 3.4 Experimental details

**Figure 4:**
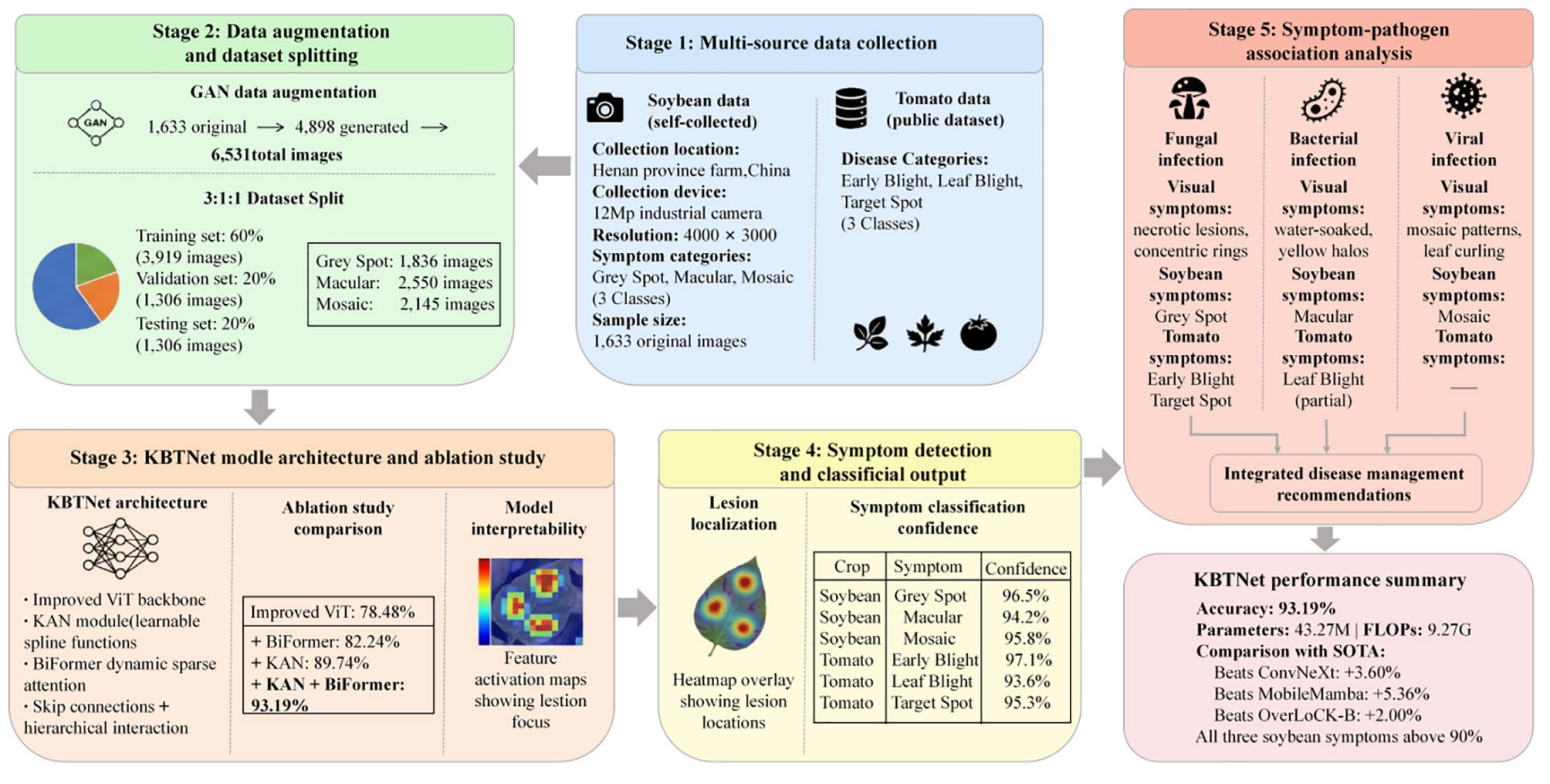
Experimental framework of crop disease symptom recognition based on our KBTNet.

**Figure 4** illustrates the experimental framework of the proposed KBTNet model, encompassing five core stages from data collection to disease diagnosis. Stage 1 involves multi-source data collection, comprising a self-collected soybean dataset (1633 images of gray spot, yellow spot, and flower and leaf symptoms) and publicly available tomato datasets (early blight, leaf blight, and target spot). Stage 2 performs data augmentation and partitioning, expanding the images to 6531 using GAN, and splitting the dataset in a 3:1:1 ratio. Stage 3 presents the KBTNet architecture and ablation experiments, achieving a complete model accuracy of 93.19%. Stage 4 outputs symptom classification with confidence exceeding 93% across all six categories. Stage 5 conducts symptom-pathogen association analysis, generating comprehensive disease management recommendations. As summarized in the bottom right, our KBTNet outperforms mainstream comparison models across multiple metrics, offering a complete technical solution for crop disease symptom recognition.

### 3.5 Data collection

**Figure 5:**
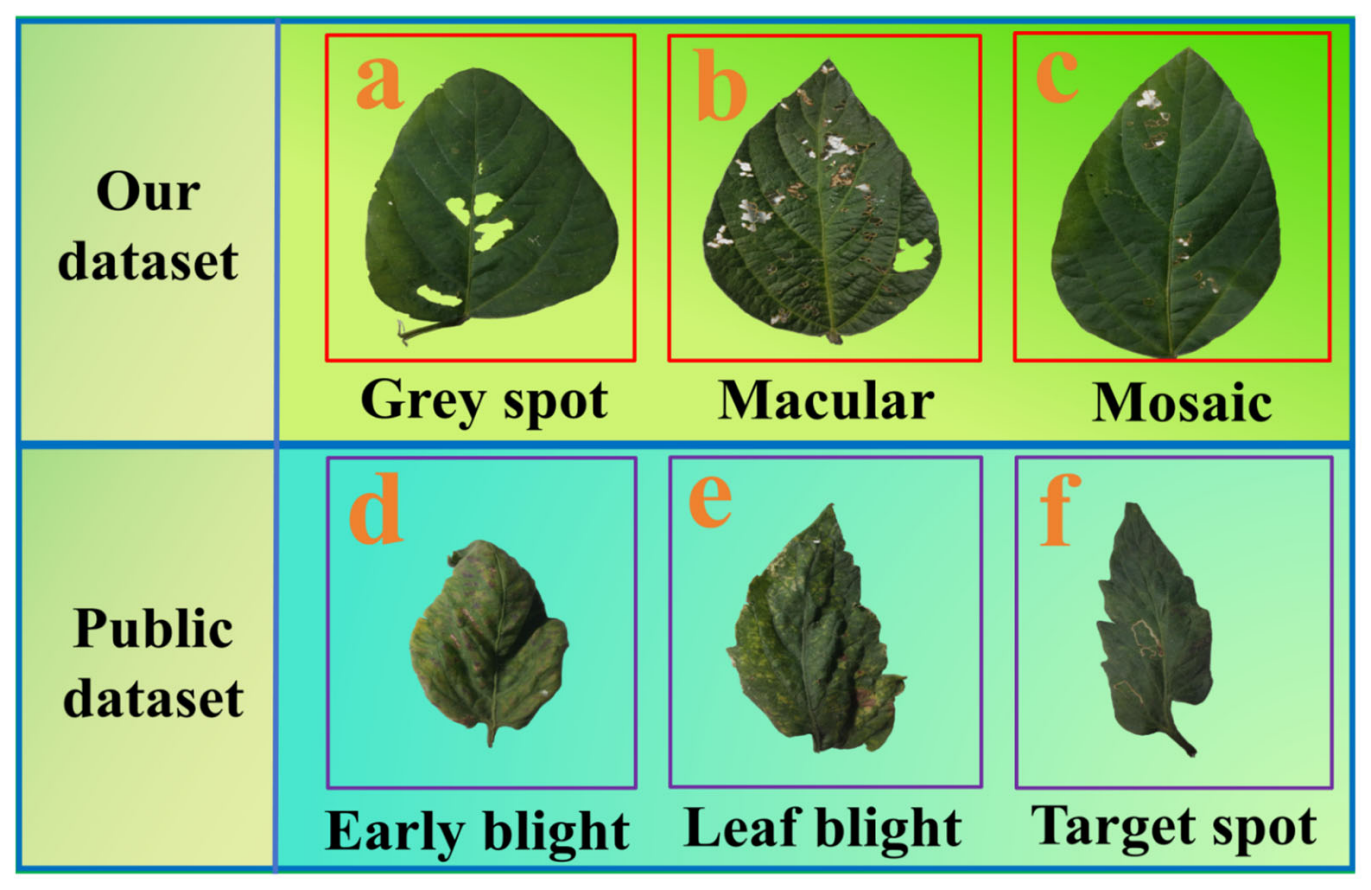
displays representative samples from proprietary and publicly available datasets. The first row presents soybean leaf samples collected in this study, while the second row shows public tomato disease samples. **(a)** Grey spot: circular to irregular lesions with chlorotic discoloration. **(b)** Macular: well-defined margins with irregular contours. **(c)** Mosaic: leaf curling and distortion. **(d)** Early blight: brown margins with gray-white centers and concentric rings. **(e)** Leaf blight: diffuse margins with yellowish halos. **(f)** Target spot: small gray-brown spots progressing to larger irregular lesions.

The experimental dataset comprises self-collected soybean disease images and a publicly available tomato disease dataset. Soybean leaf images were captured at a plantation in Henan, China, using a 12-megapixel industrial camera with 4000 × 3000 resolution, enabling detailed visualization of lesion characteristics. A dynamic range of 68 dB ensures consistent quality under varying light, while a 25 fps frame rate and IP65 rating support reliable field deployment. Representative samples are shown in **Figure 5**. For soybean, we target Grey spot, Macular, and Mosaic symptoms, visual descriptors used in pathology to differentiate fungal, viral, or bacterial pathogens. This integrated dataset supports robust evaluation of disease recognition models under diverse agricultural conditions.

During data acquisition, samples were collected at 30-minute intervals under varying weather conditions to ensure leaf freshness. All specimens were immediately sealed in preservation bags and transported to the laboratory. Image capture employed a Hikvision 12-megapixel industrial camera with 4000×3000 resolution, 68 dB dynamic range, 25 fps frame rate, and IP65 rating. The final dataset consists of 6,531 images, including 1,633 original captures and 4,898 GAN-augmented samples, covering three distinct visual disease symptom categories, such as Grey spot, Macular, and Mosaic.

Soybean leaves are susceptible to multiple diseases exhibiting distinct visual symptom patterns. Grey spot infection produces gray lesions of varying sizes, with severe cases leading to extensive chlorosis and leaf desiccation. Macular symptoms are characterized by dense, small yellow spots on the leaf surface; early-stage lesions remain discrete but may coalesce into larger patches during disease progression. Mosaic results in mottled yellow-green patterning, typically accompanied by leaf curling, growth retardation, and developmental delays. **Table 2** details the distribution of original and augmented samples.

**Table 2:** Distribution of each class on the original and augmented data.

Categories	Grey spot	Macular	Mosaic	Total
Collected data	459	638	536	1633
Generated data	1377	1912	1609	4898
Total	1836	2550	2145	6531

Tomato leaves demonstrate vulnerability to several pathogenic infections. These infections produce characteristic visual symptoms that can be recognized in leaf images. Early blight typically induces circular to sub-circular foliar lesions. Leaf blight manifests as irregular necrotic areas originating from leaf margins or tips, leading to progressive chlorosis and defoliation. Target spot presents as brown-centered lesions with distinctive concentric circles, often surrounded by chlorotic halos. These diseases collectively impair photosynthetic efficiency and physiological functions, ultimately compromising yield and quality.

This exploratory study aims to efficiently evaluate model feasibility and generalization capability. The dataset was partitioned into training, validation, and test sets following a 3:1:1 ratio. This configuration facilitates rapid iterative feedback during experimental phases while maintaining engineering relevance. Under constrained computational resources, such partitioning accelerates experimentation and enhances resource utilization efficiency. The approach aligns with established practices in exploratory deep learning research, ensuring methodological rigor and comparability. We employ a generative adversarial network Pix2Pix for data augmentation, where both the generator and discriminator consist of four convolutional layers. Training continues until the generated images visually resemble real ones in lesion morphology, color, and texture. The generated images are then merged with the original ones, and the combined dataset is randomly split in a 3:1:1 ratio, with both the validation and test sets containing generated samples. These generated images are not mere replicas of the original samples; they differ in pixel values, texture details, and lesion morphology. Therefore, including them helps enhance model generalization across diverse features without artificially inflating performance metrics due to potential similarity between generated and original data. Detailed categorical sample distributions are provided in **Table 3**.

**Table 3:** The specific distribution number of each type of data.

Categories	Training set	Validation set	Testing set	Total number
Grey spot	1102	367	367	1836
Mosaic	1287	429	429	2145
Macular	1530	510	510	2550
Total number	3919	1306	1306	6531

### 3.6 Computation equipment and software

The experimental algorithm processing platform is a personal computer equipped with an AMD Ryzen 5 5600H processor and a Radeon Graphics card. The software environment is Windows 11 ×64. The deep learning framework used is PyTorch 2.3.0, the programming language is Python 3.9.13, and the development tool is PyCharm 2024.3.1.1.

To determine the optimal hyperparameters for the proposed KBTNet model, we investigated the effects of different initial learning rates (LR), KAN learning rates, training epochs, and batch sizes on model performance. The results are presented in **Table 4**.

**Table 4:** Experimental parameter configuration under different hyperparameter settings.

Initial LR	KAN LR	Epoch	Batch size	Test loss	Test accuracy
0.001	0.0001	20	8	0.2345	89.74%
0.001	0.0001	30	8	0.1678	91.87%
0.001	0.0001	50	8	0.0740	93.19%
0.001	0.0001	100	8	0.1089	92.48%
0.0005	0.0001	50	8	0.1423	92.34%
0.001	0.0005	50	8	0.0987	92.71%
0.001	0.0001	50	16	0.1189	92.56%

**Table 4** reports that different network parameter settings yield varying test results. Among all compared configurations, the optimal one used an initial learning rate of 0.001, a KAN learning rate of 0.0001, 50 training epochs, and a batch size of 8, achieving the highest test accuracy of 93.19%. Relative to the worst configuration (initial learning rate=0.0001, batch size=8, accuracy =91.98%), this represents a 1.21% improvement. Compared with the suboptimal configuration (initial learning rate=0.001, KAN learning rate=0.0005, epoch=50, batch size 8, and accuracy= 92.71%), the improvement is 0.48%. Accordingly, we adopt the optimal configuration.

## 4 Results & discussions

### 4.1 Model improvement results

**Figure 6:**
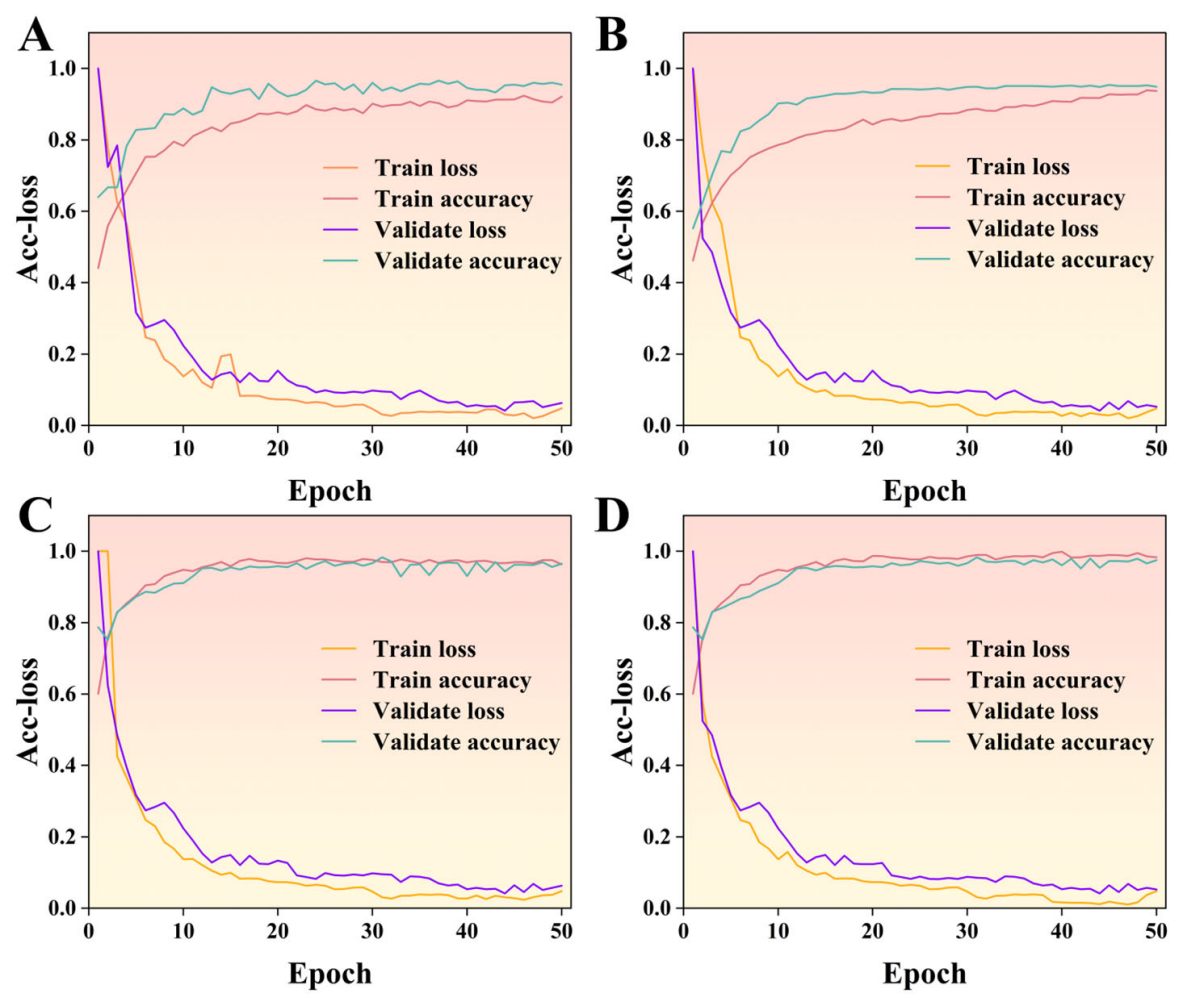
Accuracy and loss curve. **(A–D)** represent the accuracy and loss curves on the training and validation sets for Improved ViT, Improved ViT+BiFormer, Improved ViT+KAN, and Improved ViT+KAN+BiFormer, respectively.

The model underwent iterative training on both the training and validation sets. The changes in accuracy and loss values on the training and validation sets across different iterations are in accuracy and loss values on the training and validation sets across different iterations are illustrated in **Figure 6**.

As revealed in **Figure 6**, the proposed Improved ViT+KAN+BiFormer model achieves superior performance in soybean leaf disease symptom recognition, demonstrating the highest accuracy, the most rapid loss reduction, and the most stable convergence. Ablation studies indicate that incorporating the KAN module yields substantially greater performance improvement on the validation set compared to using BiFormer alone, underscoring KAN’s critical role in feature enhancement. The synergistic combination of KAN and BiFormer enhances both discriminative capability and training efficiency without significantly increasing the parameter count. This integrated framework presents an effective and innovative solution for intelligent soybean disease symptom identification.

### 4.2 Model evaluation on soybean and tomato crops

**Figure 7:**
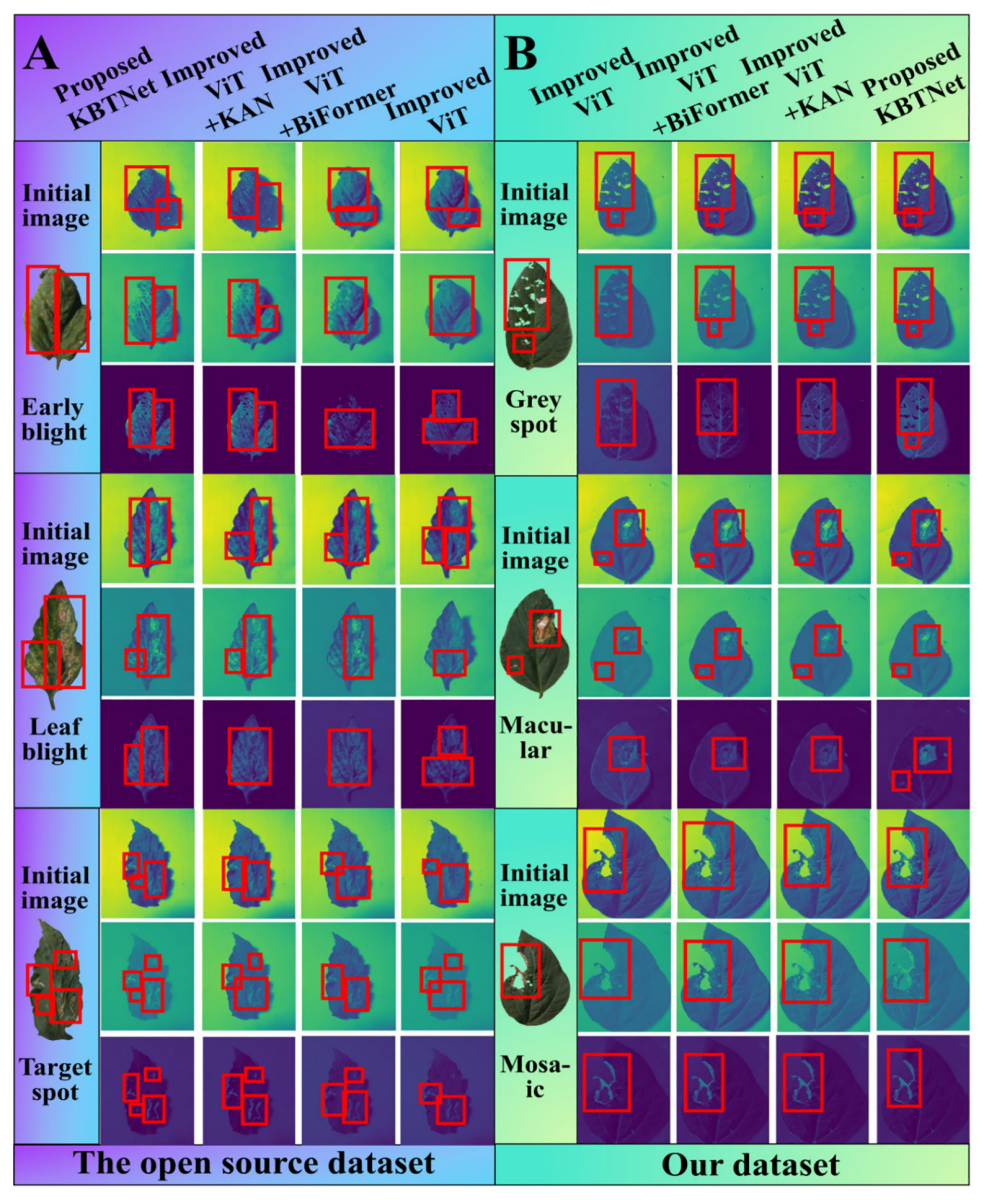
visualizes disease symptom-related features in soybean and tomato leaves. **(A)** shows feature extraction by Improved ViT and its ablated variants on public data featuring Early blight, Leaf blight, and Target Spot. **(B)** presents corresponding results on proprietary data covering Grey spot, Macular, and Mosaic.

To further verify the effectiveness of the proposed ablation method for extracting features of leaf disease symptoms, we visualized the feature extraction maps corresponding to the symptomatic regions of leaves under different ablation experimental methods. The visualized heatmap results are shown in **Figure 7**.

**Figure 7** demonstrates that the baseline Improved ViT generates feature heatmaps with scattered activations, exhibiting weak responses in lesion regions and limited sensitivity to small lesions. Integrating the KAN module substantially intensifies and consolidates activations within diseased areas, reflecting its enhanced nonlinear representation of pathological structures. Adding the BiFormer module further sharpens lesion boundary localization through bidirectional attention mechanisms. The combined KAN-BiFormer architecture produces highly focused activations across both prominent and subtle lesions, illustrating how feature enhancement and spatial modeling synergistically extract discriminative multi-scale disease symptoms.

**Figure 8:**
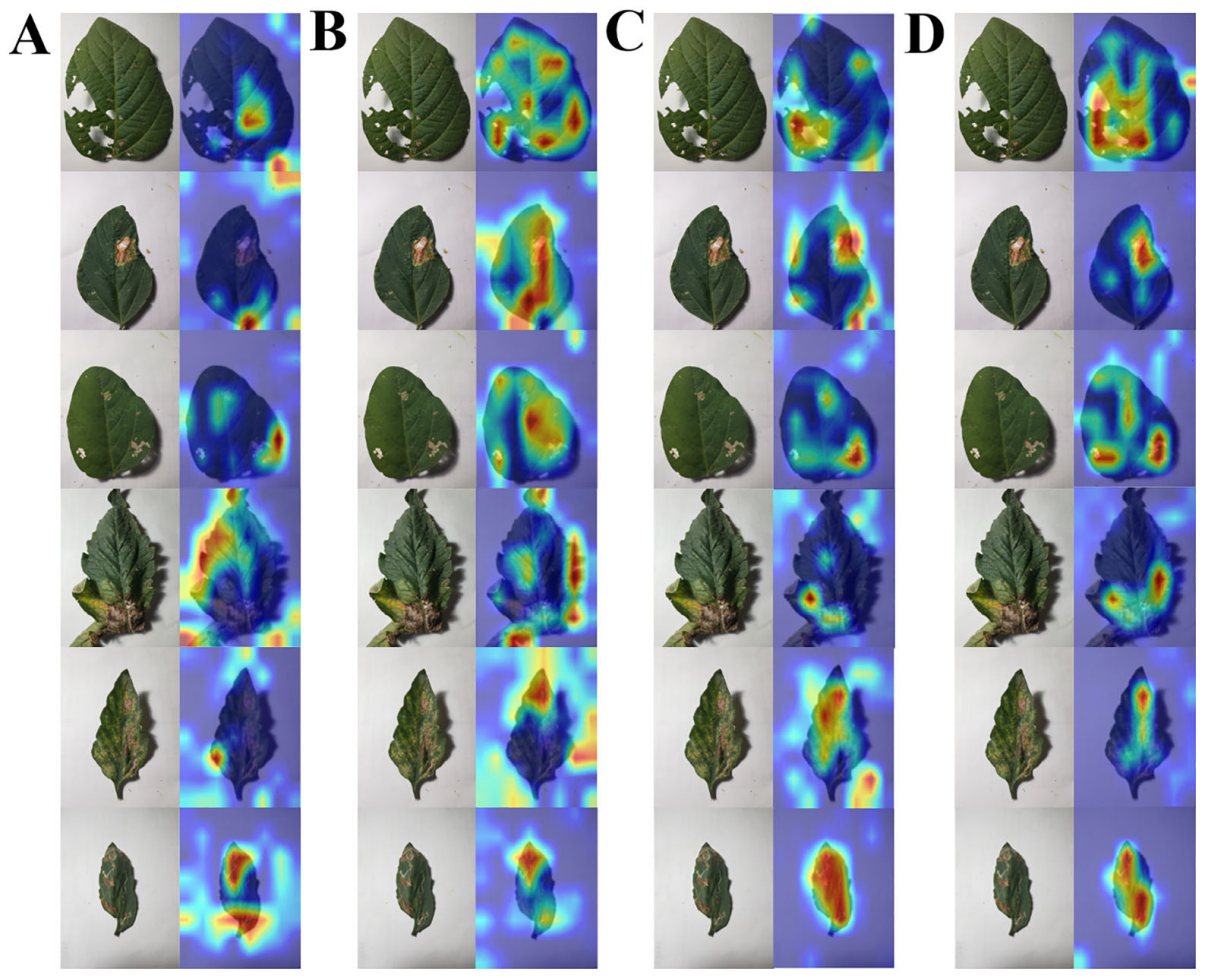
Grad CAM heatmaps of soybean leaf disease feature extraction under different ablation experiments. **(A)** is the improved ViT. **(B)** is the improved ViT+KAN. **(C)** is the improved ViT+BiFormer. **(D)** is the improved ViT+KAN+BiFormer.

We introduce three key architectural improvements: a lightweight Vision Transformer for efficient inference, BiFormer’s dynamic sparse attention for localized feature extraction, and KAN modules for enhanced nonlinear representation in **Figure 8**.

**Figure 8** demonstrates that the proposed Improved ViT+KAN+BiFormer model generates the most focused and discriminative feature responses across all six visual disease symptom categories, precisely highlighting key pathological regions. Comparative analysis reveals limitations in other variants: Improved ViT and Improved ViT+BiFormer exhibit scattered activations with background noise in Mosaic and Early blight, while Improved ViT+KAN shows limited cross-class generalization despite enhanced local feature extraction. These results confirm that integrating KAN with BiFormer significantly improves both discriminative capability and region-specific localization, yielding a more reliable visual framework for leaf disease visual symptom identification.

**Figure 9:**
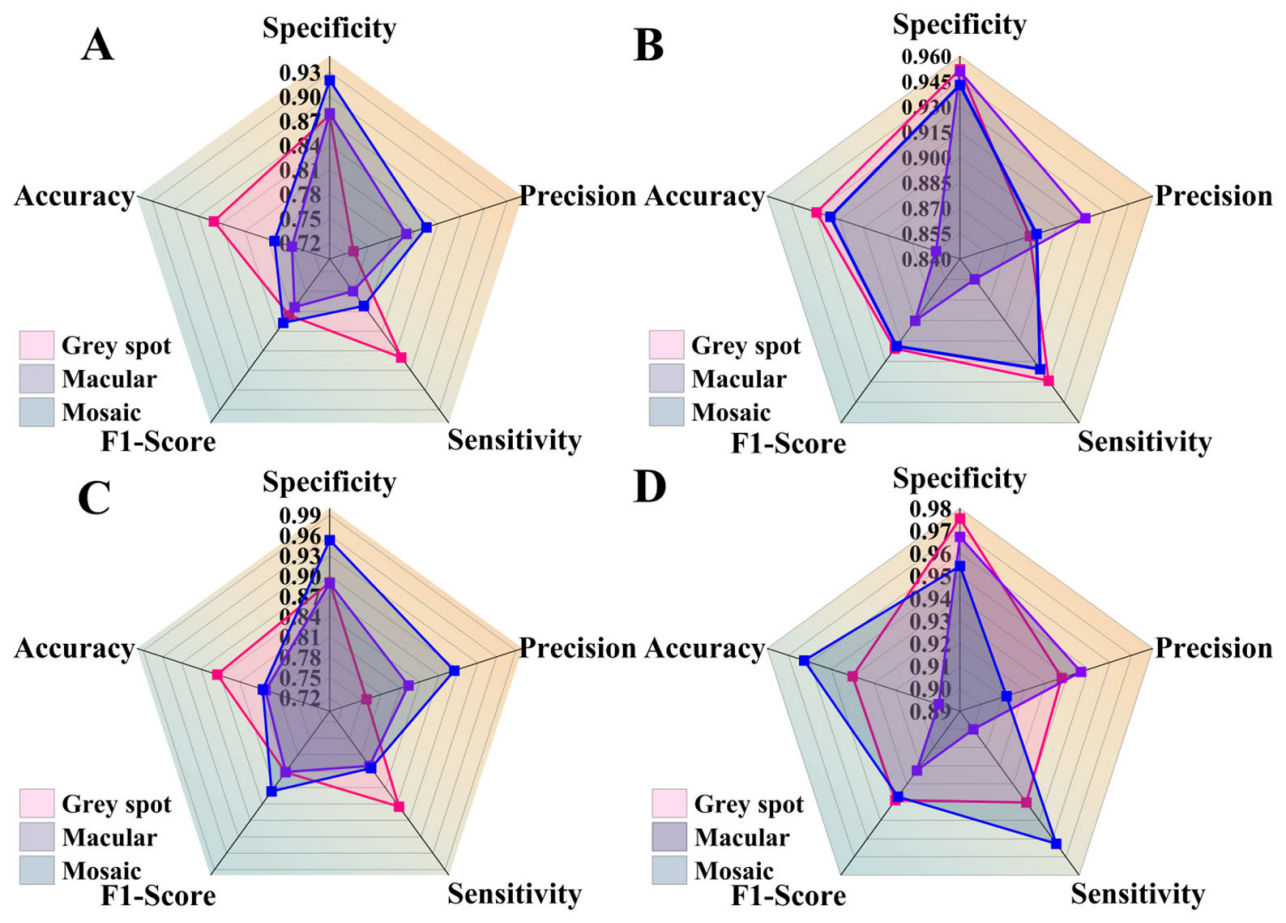
Comparison of evaluation metrics for different models. **(A–D)** represent radar charts of five evaluation metrics for Improved ViT, Improved ViT+KAN, Improved ViT+BiFormer, and Improved ViT+KAN+BiFormer, respectively.

Radar charts offer standardized visualization of multi-dimensional metrics through axes radiating from a central point, forming polygonal shapes from connected variables. We present ablation experiment results for three soybean leaf disease symptom categories in **Figure 9**.

As elucidated in **Figure 9**, the proposed model achieves strong performance across all evaluation metrics. It attains a 3.32% higher F1-score and 1.09% greater accuracy in grey spot recognition compared to the Improved ViT+KAN baseline. The integration of KAN and BiFormer enables the model to surpass all alternative configurations across all five metrics, exceeding 90% for all three disease types while maintaining superior accuracy, robustness, and inter-class balance. These findings demonstrate that our modular co-design strategy effectively enhances crop disease identification.

**Figure 10:**
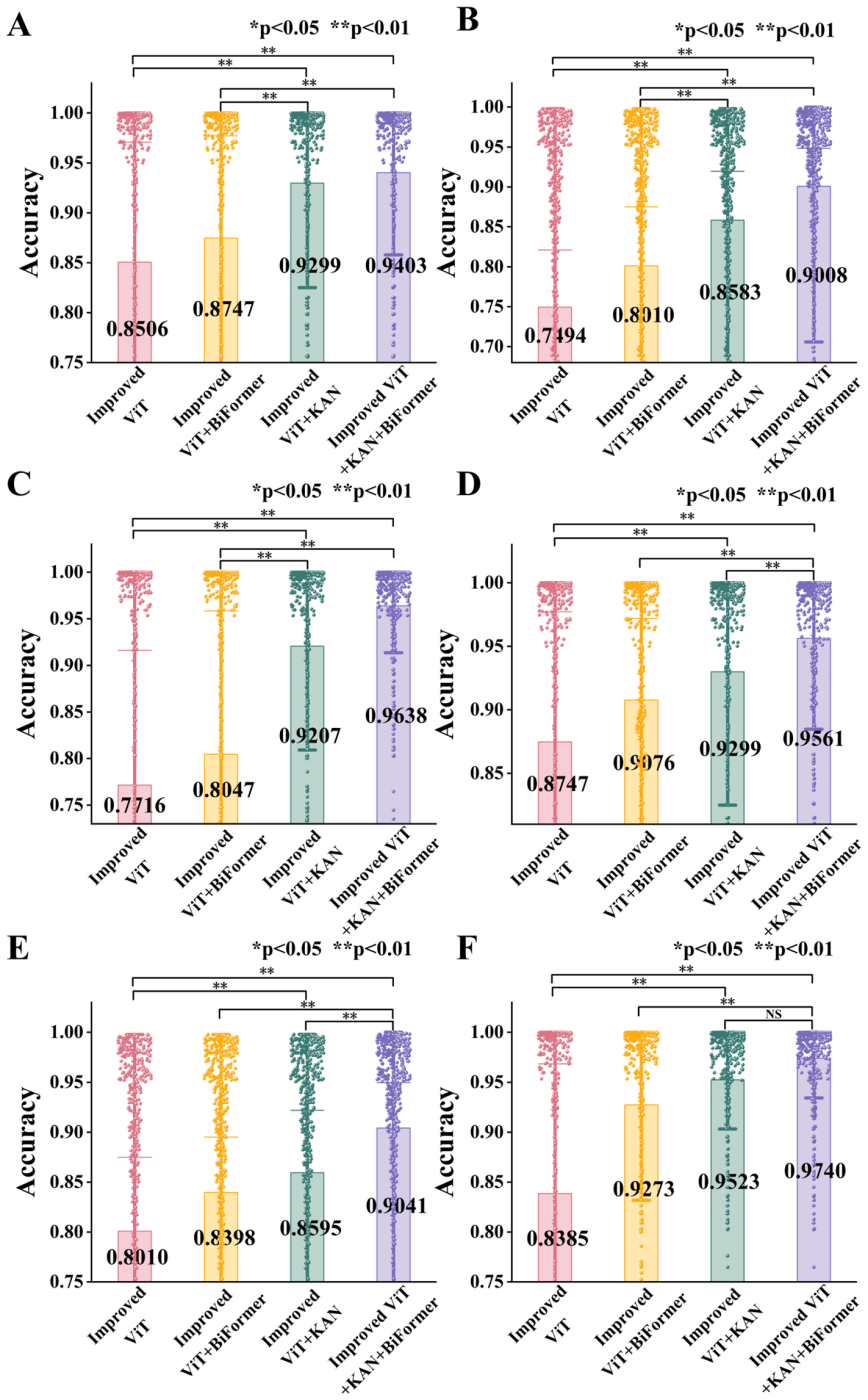
presents accuracy distributions. The proposed method achieves average accuracies exceeding 85% for Grey spot **(A)**, 74% for Macular **(B)**, 77% for Mosaic **(C)**, 87% for Early blight **(D)**, 80% for Leaf blight **(E)**, and 83% for Target spot **(F)**, with all methods surpassing 90% mean accuracy. * Indicates a p-value less than 0.05. ** Indicates a p-value less than 0.01.

Bar-scatter plots integrate bars, scatter points, and significance markers to visualize data distributions, central tendencies, and statistical differences. **Figure 10** employs this method to display accuracy distributions across models for soybean disease classification, where bars show means, scatters indicate data distribution, and asterisks denote statistical significance.

**Figure 10** presents confidence distributions for three proprietary dataset diseases (A–C) and three public dataset diseases (D–F). The complete Improved ViT+KAN+BiFormer model demonstrates superior overall recognition performance compared to ablated versions. It achieves accuracy improvements of approximately 1.0%, 6.5%, and 8.8% over Improved ViT+KAN, Improved ViT+BiFormer, and baseline Improved ViT, respectively. Enhanced performance on Mosaic and Target Spot diseases indicates the integrated architecture’s improved discrimination of subtle pathological features. These results confirm that combining KAN with BiFormer enhances feature representation and classification stability for robust soybean disease recognition.

**Figure 11:**
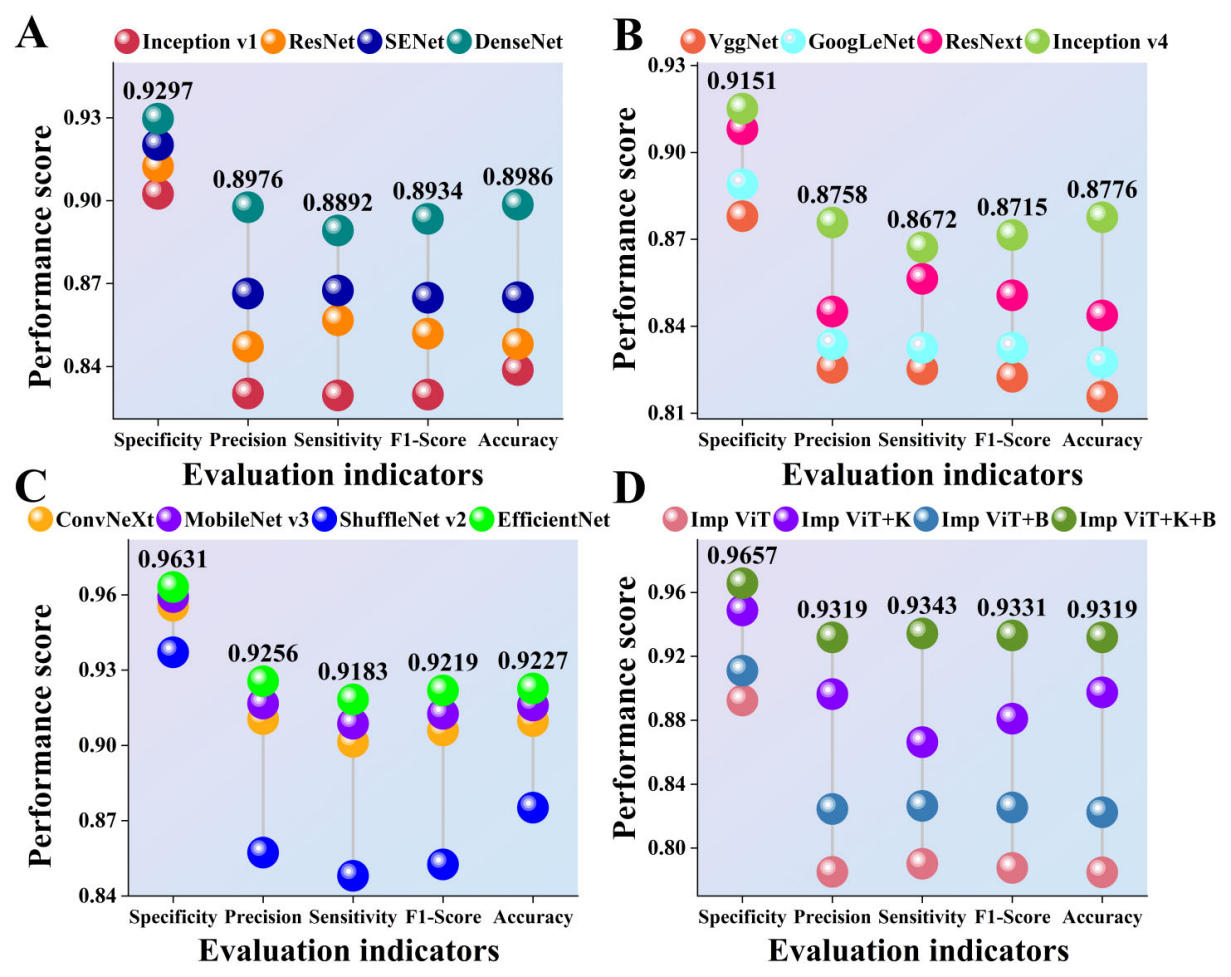
compares performance metrics across four models: original **(A)**, baseline **(B)**, lightweight **(C)**, and our proposed approach **(D)**. Labels denote Improved ViT (Imp ViT), Improved ViT+KAN (Imp ViT+K), Improved ViT+BiFormer (Imp ViT+B), and Improved ViT+KAN+BiFormer (Imp ViT+K+B).

We evaluate soybean disease classification models using parallel coordinates plots, representing five metrics, including Specificity, Sensitivity, Precision, F1-score, and Accuracy. This visualization reveals relative strengths and performance balance across models, guiding refinement. The results are presented in **Figure 11**.

**Figure 11** illustrates the superior performance of the Improved ViT+KAN+BiFormer model, which outperforms Inception v4 by 1.94% in specificity and exceeds EfficientNet by 0.63% in precision and 1.6% in sensitivity. Across key evaluation metrics, the integrated architecture consistently surpasses conventional models by 2–4%. This performance gain is driven by the KAN module’s non-linear enhancement of subtle lesion features and the BiFormer module’s dynamic focus on symptom regions, which collectively strengthen the model’s feature discrimination capability. These results confirm the enhanced effectiveness of the proposed approach for soybean leaf disease recognition.

### 4.3 Proposed improved model vs other traditional models

**Figure 12:**
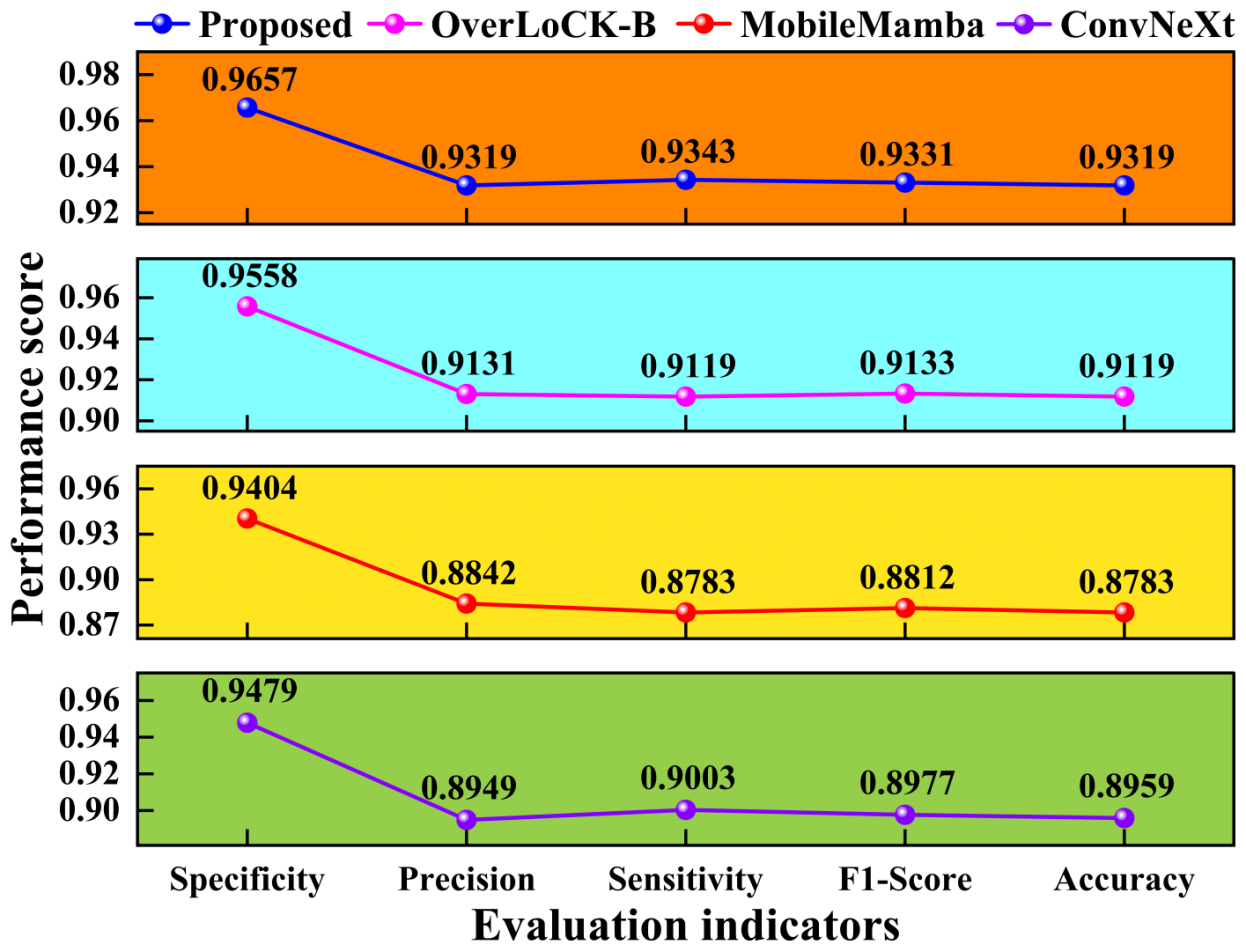
Comparison of evaluation metrics between the proposed model and SOTA models, including OverLoCK-B, MobileMamba, and ConvNeXt.

We compare the proposed method against advanced models, including ConvNeXt and MobileMamba, using an independent soybean disease dataset. Evaluation covers five metrics (i.e., Recall, Precision, Specificity, F1-score, and Accuracy) are displayed in **Figure 12**.

**Figure 12** demonstrates that the proposed Improved ViT+KAN+BiFormer model achieves superior performance across all five evaluation metrics, surpassing all four ablated variants. It improves both Precision and F1-score by approximately 2.0% compared to the OverLoCK-B model, confirming that the effective integration of KAN and BiFormer enhances feature learning and generalization capabilities. These results validate our model’s superior accuracy and robustness for soybean leaf disease recognition.

**Figure 13:**
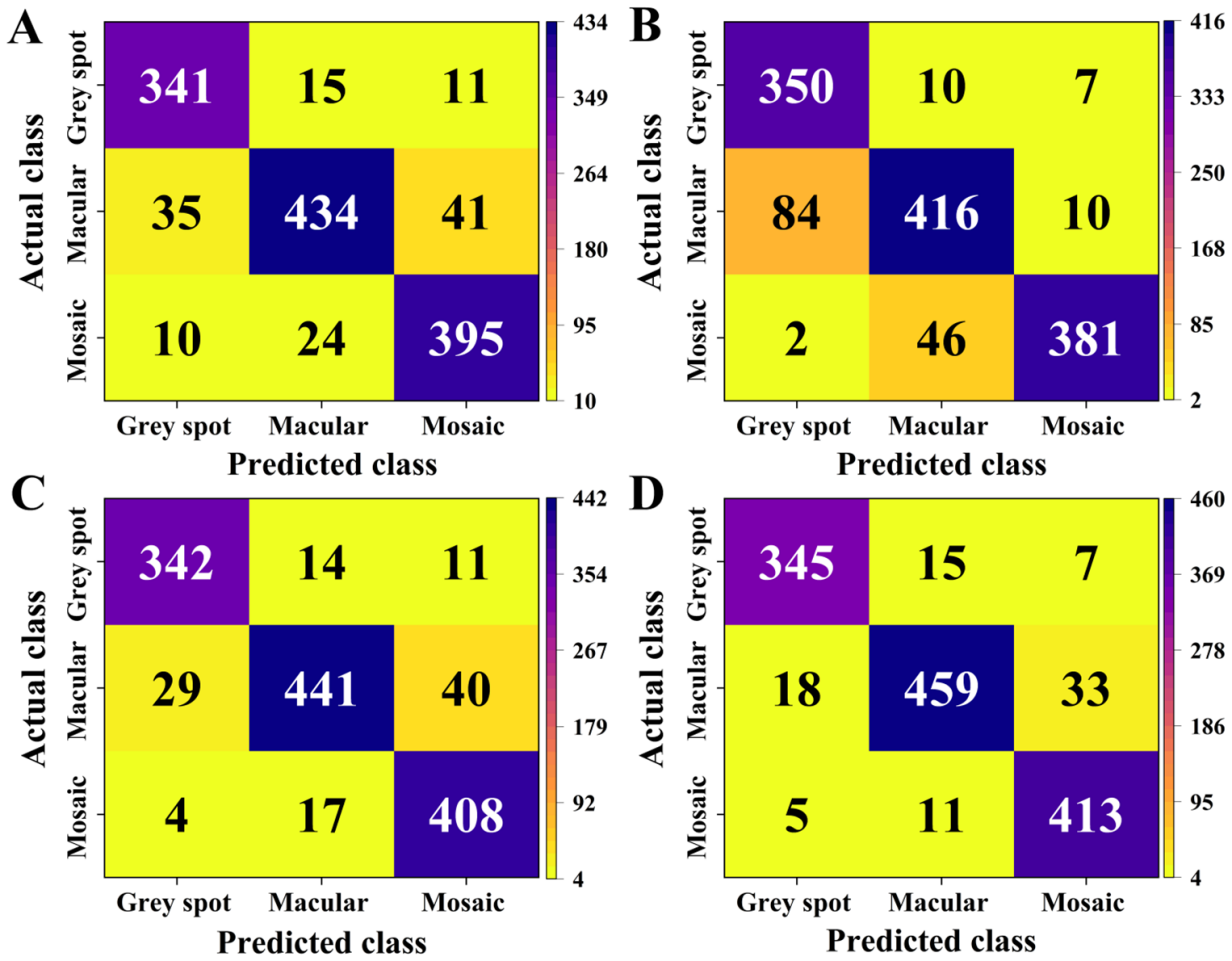
Confusion matrix classification results of different models. **(A–D)** are the distribution of classification results for ConvNeXt, MobileMamba, OverLoCK-B, and the proposed method, respectively.

The confusion matrix serves as a key tool for evaluating classification model performance in deep learning, revealing the alignment between predicted and actual labels and helping to assess the model’s discriminative capability. It enables systematic comparison of different models, identification of category-specific performance patterns, and supports targeted model refinement. To visually compare classification results for three soybean leaf diseases, we present confusion matrices for each model in **Figure 13**.

**Figure 13** presents the confusion matrices comparing classification performance across different models. Panels A, B, C, and D correspond to ConvNeXt, MobileMamba, OverLoCK-B, and the proposed model, respectively. The overall classification accuracy is 89.59% for ConvNeXt, 87.83% for MobileMamba, 91.19% for OverLoCK-B, and 93.19% for our method. Compared to MobileMamba and OverLoCk-B, the proposed approach achieves improvements of 5.36% and 2.0%, respectively, while yielding the fewest misclassifications. This superior performance is attributed to the synergistic integration of KAN and BiFormer, which enhances the extraction of subtle lesion features and effectively reduces inter-class misclassification. However, the model exhibits slightly lower performance in identifying macular disease, likely due to visual similarities between symptoms such as yellowing and curling induced by advanced lesions and those of other diseases, leading to residual confusion. Collectively, these results demonstrate the effectiveness of our method for soybean leaf disease classification.

In addition to accuracy, we also compared the parameter count and computational complexity of each model in **Table 5**. As can be seen in **Table 5**, ConvNeXt-B has 89 M parameters and 15.4 GFLOPs, OverLoCK-B has 95 M parameters and 16.7 GFLOPs, and MobileMamba has 4.8 M parameters and 0.19 GFLOPs. Our proposed model achieves 43.27 M parameters and 9.27 GFLOPs, outperforming ConvNeXt-B and OverLoCK-B in both metrics while attaining the highest recognition accuracy of 93.19%. Although MobileMamba has lower resource consumption, its accuracy is considerably lower at 87.83%.

**Table 5:** Comparison of competitive model complexity.

Models	Parameters (M)	FLOPs (G)	Accuracy
ConvNeXt-B	89	15.4	89.59%
MobileMamba	4.8	0.19	87.83%
OverLOCK-B	95	16.7	91.19%
Proposed	43.27	9.27	93.19%

These results indicate that the proposed method achieves higher recognition accuracy with lower parameter count and computational complexity than ConvNeXt-B and OverLoCK-B, confirming its favorable balance between accuracy and efficiency. Based on the comparisons in **Figures 12**, **13**, **Table 5**, our method outperforms existing mainstream models in both recognition performance and model lightweighting.

### 4.4 Model testing on diverse agricultural crops

To evaluate the performance of the proposed method on different plant leaf disease datasets, we tested it using multiple open-source datasets. The results are presented in **Table 6**. These experiments were conducted independently on each dataset, demonstrating the adaptability of the model to different data sources. However, rigorous cross-domain generalization experiments, such as training on one dataset and testing on another, have not yet been performed. Rigorous validation of domain generalization remains an important direction for future work.

**Table 6:** Comparison of test results for different plant leaf diseases.

Dataset names	Recall	Precision	F1-score	Specificity
Our own soybean leaf disease data	93.43%	93.19%	93.31%	96.57%
Plant Pathology Apple Dataset	93.38%	93.27%	93.32%	96.71%
New Plant Diseases Dataset (Potato)	93.32%	93.25%	98.28%	96.54%
PlantifyDr Dataset (Corn)	93.35%	93.04%	93.19%	96.45%
Rice Diseases Image Dataset	93.37%	93.24%	93.30%	96.52%

**Table 6** demonstrates consistent cross-domain performance across four public plant disease datasets. All metrics remain stable, with Recall varying within 0.11% and Precision differing by only 0.23% between datasets. The F1-score reaches 98.28% on the Potato dataset versus 93.31% on proprietary soybean data, while Specificity peaks at 96.71% on the Apple dataset. These results illustrate that the model maintains good stability across different data sources, indicating its potential for multi-crop disease identification. Many plant diseases present common visual symptoms, such as necrotic spots, chlorosis, and discoloration of flowers and leaves. By focusing on these fundamental symptom patterns rather than crop-specific features, the model achieves stable cross-crop performance.

We collected literature on plant leaf disease identification published in the past three years, and the results are sketched in **Table 7**.

**Table 7:** Comparison between the proposed method and the latest literature.

Ref., Year	Methods	Dataset size	Number of classes	Accuracy
Yu et al. (2023)	RANet18	4301	3	96.50%
Deng et al. (2023)	MC-UNet	4622	4	91.32%
Zou et al. (2024)	ECVNet	12972	30	92.46%
Bachhal et al. (2024)	PRFSVM	6718	6	96.67%
Bhavani (2025)	CAE	1166	5	92.00%
Bouacida et al. (2025)	Inception	54305	38	94.04%
Proposed	KBTNet	6531	3	93.19%

**Table 7** demonstrates that the proposed method achieves 1.87% higher accuracy than Deng et al.’s MC-UNet and 1.19% higher accuracy than Bhavani’s Convolutional Autoencoder. Although RANet18 shows a 3.31% accuracy advantage, our model maintains competitive performance compared to existing approaches and fulfills the fundamental requirements for soybean leaf disease identification.

**Figure 14:**
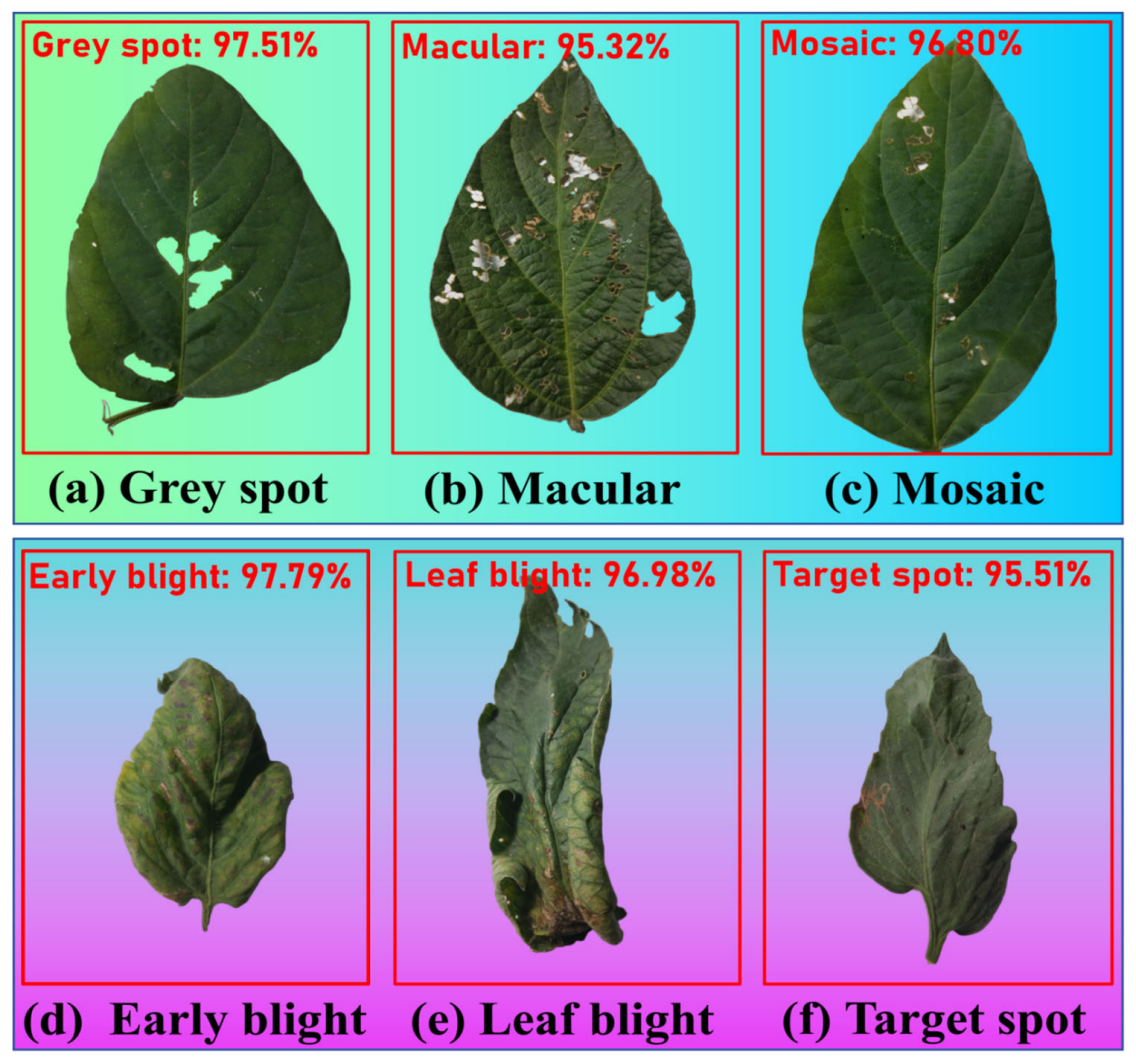
Prediction results. The first line is our dataset, and the second line is the public dataset. **(a)**, **(b)**, and **(c)** are our dataset. **(d)**, **(e)**, and **(f)** are the public dataset.

We randomly selected six samples from our and public test sets for testing, as provided in **Figure 14**, and the confidence level of each recognition probability was above 95%.

## 5 Implications for precision agriculture and field diagnostics

### 5.1 Edge deployment potential for real-time diagnostics

We present a novel computer vision and deep learning-aided crop leaf disease symptom recognition model integrating the Kolmogorov-Arnold network (KAN) and BiFormer modules into a Vision Transformer architecture. The model achieves 93.19% accuracy on a three-disease symptom dataset, outperforming established models including EfficientFormer, MobileOne-S0, and OverLoCK-B. With 43.27 million parameters, it meets edge deployment requirements. The core constraints of edge deployment are the number of model parameters and computational complexity. Compared with the original ViT (85.80M parameters, 16.86G FLOPs), our KBTNet reduces parameter count by 49.6% and FLOPs by 45.0%, meeting the basic deployment requirements of edge devices such as Jetson Nano and laying a foundation for future deployment and testing on actual edge hardware. We evaluate five optimized variants on hardware with 16 GB of memory and Radeon Graphics, measuring parameters, FLOPs, inference speed, and accuracy. Detailed results appear in **Table 8**.

**Table 8:** Comparison of resource consumption parameters for different models.

Model version	Params (M)	FLOPs (G)	Speed (s)	Accuracy
ViT	85.80	16.86	1.00	58.98%
Improved ViT	48.57 (↓37.23)	8.49 (↓8.37)	1.99 (↑0.99)	78.48%(↑19.50%)
Improved ViT+KAN	46.27 (↓39.53)	11.64 (↓5.22)	1.45 (↑0.45)	89.74%(↑30.76%)
Improved ViT+BiFormer	47.70 (↓38.10)	9.91 (↓6.95)	1.70 (↑0.70)	82.24%(↑23.26%)
Improved ViT+KAN+BiFormer	43.27 (↓42.53)	9.27 (↓7.59)	1.82 (↑0.82)	93.19%(↑34.21%)

↓ indicates a decrease.

↑ indicates improvement.

Experimental results demonstrate that the improved ViT+KAN+BiFormer architecture achieves optimal performance with 43.27 million parameters. The enhanced ViT maintains competitive inference speed while reducing computational complexity. The KAN module reduces parameters by 2.30 million but increases computational cost by 3.15 GFLOPs, extending inference time by 0.54 seconds. Bi-Directional integration moderately increases FLOPs while substantially enhancing representational capacity. All optimized variants operate within practical inference constraints, effectively balancing computational efficiency and speed. This design demonstrates particular suitability for resource-limited edge environments. Future work will employ quantization techniques to further optimize performance for embedded systems.

In the future, we plan to embed the proposed deep learning model into our developed edge mobile device, Jetson Nano, for real-time ground detection of plant leaf disease symptoms. This approach shows strong potential for edge deployment within agricultural production systems. Through structural modifications to embedding dimensions and attention mechanisms in ViT, we achieve significant reductions in both parameter count and computational complexity. While incorporating KAN and BiFormer moderately increases model size, it substantially enhances recognition accuracy while maintaining manageable computational demands. The optimized architecture operates efficiently on resource-constrained edge devices, preserving lightweight characteristics while enabling rapid image processing for in-field disease symptom diagnostics. It meets stringent requirements for real-time response and power efficiency in edge environments, effectively balancing accuracy with operational performance. This adaptability to diverse mobile deployment scenarios provides practical technical support for implementing edge intelligence in crop disease monitoring systems.

### 5.2 Integration with UAV and UGV systems

The proposed KBTNet model is well-suited for integration with unmanned aerial vehicles.

(UAVs) and unmanned ground vehicles (UGVs). Through architectural improvements to the Vision Transformer, the model achieves a lightweight design with 43.27 million parameters and a computational complexity of 9.27 GFLOPs while maintaining a recognition accuracy of 93.19%. This configuration satisfies the fundamental requirements for edge deployment, enabling its deployment on mobile platforms such as agricultural drones and inspection robots for autonomous field surveillance and real-time disease monitoring.

Following integration, drones can rapidly acquire crop canopy images and identify the spatial distribution of disease symptoms in real time, while UGVs can conduct near-ground observations to capture detailed foliar features. This collaboration enables a monitoring framework that combines wide-area aerial screening with ground-based precise confirmation, thereby enhancing early warning capabilities and disease control efficiency. Future work may focus on further optimizing inference speed through model quantization and hardware acceleration to satisfy the real-time constraints of higher-frame-rate video streams.

### 5.3 Mobile version for small-scale farmers

Developing mobile applications based on the KBTNet model holds significant potential for addressing the practical needs of smallholder farmers. The model’s optimization in terms of parameter count and computational efficiency facilitates its deployment on widely accessible devices such as smartphones. Further model compression through techniques like knowledge distillation and quantization-aware training can enable offline operation on mobile platforms, thereby reducing dependence on network connectivity.

This system enables real-time field imaging, rapid identification of disease symptoms, and intelligent delivery of management recommendations, thereby assisting smallholder farmers in monitoring crop health conditions even without access to professional technical support. In addition, it integrates recognition capabilities for multiple crops, including soybeans, tomatoes, and potatoes, to address the diverse agricultural needs associated with small-scale farming. This low-barrier, easily deployable technological solution helps reduce disparities in access to digital agricultural services and facilitates the practical implementation of smart farming technologies at the grassroots level.

## 6 Limitations and future studies

Despite the promising results demonstrated by the proposed KBTNet framework for plant disease symptom recognition, several limitations of this study should be acknowledged.

### 6.1 Limitations

First, although augmented, the primary dataset used for model development and evaluation remains limited in scale, comprising 6,531 images that cover only three visual symptom categories for soybean (Grey spot, Macular, and Mosaic) and three for tomato. This narrow scope may not adequately represent the wide spectrum of economically significant diseases, such as soybean rust, downy mildew, or bacterial pustules, which exhibit distinct visual characteristics. Consequently, the model’s generalization capability to a broader range of pathogens and crop species requires further validation. Second, while the model was tested on multiple public datasets to assess cross-crop adaptability, the images in these datasets, including our own, were primarily captured under controlled or semi-controlled conditions. The robustness of the model in real-world agricultural settings, which involve complex challenges such as variable illumination, occlusion from overlapping leaves, complex soil backgrounds, and the simultaneous presence of multiple diseases or pests on a single leaf, has not been extensively evaluated. Performance may degrade under such high noise and dynamic field conditions. Third, despite improvements in computational efficiency for edge deployment, the model still presents challenges. With 43.27 million parameters, its inference speed on truly resource-constrained devices, such as low-power microcontrollers or older generation drones, may not yet satisfy the requirements for real-time, high-throughput processing. The trade-off between the accuracy gains from the KAN and BiFormer modules and the resulting computational overhead necessitates further hardware-specific optimization. Lastly, the current study focuses on image-based analysis of static visual symptoms and does not integrate other crucial data modalities that could enhance diagnostic accuracy and provide a more holistic view of plant health. Factors such as environmental conditions, including temperature and humidity, temporal disease progression, and plant genotypic information are not considered, which limits the system’s potential for predictive modeling and precision management. In addition to the above limitations, the validation of model generalization also has shortcomings. This study demonstrates model adaptability to different data sources by testing on multiple publicly available datasets. However, these experiments do not constitute strict domain generalization testing. Rigorous cross-domain generalization experiments, such as training on one dataset and testing on another (e.g., training on soybean data and testing directly on apple data without fine-tuning), have not yet been conducted. Therefore, current conclusions regarding cross-crop generalization should be interpreted with caution.

### 6.2 Future work

To address these issues and advance this research toward a practical, field-deployable solution, future work will be directed along several key paths. We plan to expand the dataset to include more disease categories relevant to agriculture and employ augmentation techniques to improve sample diversity. Model robustness will be strengthened through attention refinement and adversarial training tailored to horticultural imagery. Furthermore, we intend to extend this system toward a multimodal plant disease monitoring platform that integrates hyperspectral imaging and environmental sensors. Such an approach could improve diagnostic stability across horticultural growing seasons and environmental conditions. By incorporating spatiotemporal modeling and scalable edge computing, this framework could support dynamic disease progression tracking and provide timely decision support for integrated disease management in soybean and other horticultural crops. Finally, model compression along with hardware-aware optimization will be applied to accelerate edge inference for field deployment. For inference speed testing on edge hardware, we plan to deploy the model on edge devices such as the Jetson Nano. This will involve model conversion and TensorRT acceleration, evaluation of inference latency and throughput under different batch sizes, and application of INT8 or FP16 quantization to assess the tradeoff between accuracy and speed. Real-time detection capability will also be validated under actual field conditions.

In addition, we plan to extend the proposed KBTNet framework to tasks such as disease severity estimation and lesion segmentation. Specifically, we will use the attention weights of the BiFormer module to generate heatmaps for estimating lesion area proportion or classifying severity levels (e.g., mild, moderate, and severe). We will also combine the nonlinear feature representation capability of the KAN module with a decoder architecture such as U Net to achieve segmentation of diseased areas at the pixel level. These extensions are explicitly part of our future research plans.

## 7 Conclusions

In this work, we developed a vision-based diagnostic framework for plant leaf disease symptom identification by integrating an enhanced Vision Transformer with a Kolmogorov-Arnold network and a BiFormer dynamic attention mechanism. The proposed KBTNet architecture introduces three key innovations. First, a lightweight Vision Transformer backbone is optimized through reduced embedding dimensions and fewer transformer layers to minimize computational redundancy. Second, a Kolmogorov-Arnold network replaces conventional multilayer perceptrons with learnable spline-based univariate functions, enabling superior nonlinear feature representation for capturing subtle variations in lesion textures, discoloration patterns, and spot morphologies. Third, a BiFormer dynamic sparse attention mechanism employs region-aware routing to concentrate computational resources on symptom salient regions while suppressing irrelevant background interference. This synergistic design achieves effective multi-scale feature extraction by balancing global context modeling with localized symptom focus through hierarchical interaction mechanisms and skip connections. Experimental results demonstrated that the framework achieved 93.19 percent classification accuracy for three key soybean disease symptoms: Grey spot, Macular, and Mosaic, outperforming state-of-the-art models including ConvNeXt (by 3.60 percent), MobileMamba (by 5.36 percent), and OverLoCK B (by 2.00 percent). Comprehensive ablation studies confirmed that the KAN module contributed an accuracy improvement of 11.26% over the baseline Improved ViT, while the BiFormer module added 3.76%, with their combination yielding synergistic gains in feature discriminability and classification stability. The proposed model demonstrated stable performance on multiple public datasets, including tomato, apple, potato, corn, and rice, indicating its potential for disease recognition across different crops. However, these results come from independent evaluations of each dataset rather than rigorous cross-domain generalization experiments, such as transfer learning from a source domain to a target domain. This limitation will be addressed in future research. With 43.27 million parameters and 9.27 GFLOPs computational complexity, the model achieves an optimal balance between accuracy and efficiency. demonstrating strong potential for deployment on resource-constrained edge devices such as agricultural drones and portable field detectors. This work provides a scalable and efficient solution for intelligent crop disease monitoring and supports the integration of vision-based diagnostics into agricultural management systems.

## Data Availability

The original contributions presented in the study are included in the article/**Supplementary Material**. Further inquiries can be directed to the corresponding authors.
