# Supplementary material for: Integrating Kolmogorov-Arnold networks and sparse attention for robust visual plant disease symptom identification across diverse agricultural crops
Source: Front Plant Sci. 2026 Jul 2;17:1855159. doi: 10.3389/fpls.2026.1855159 (PMC13373955; doi:10.3389/fpls.2026.1855159)
Supplement: Supplementary file 1 [file DataSheet1.pdf]

A correlation heatmap visualizes variable relationships through a color-coded matrix, with colors indicating Pearson correlation coefficients ranging from -1 to 1. We employ this method to analyze interdependencies among model performance metrics in Appendix Fig. 1.

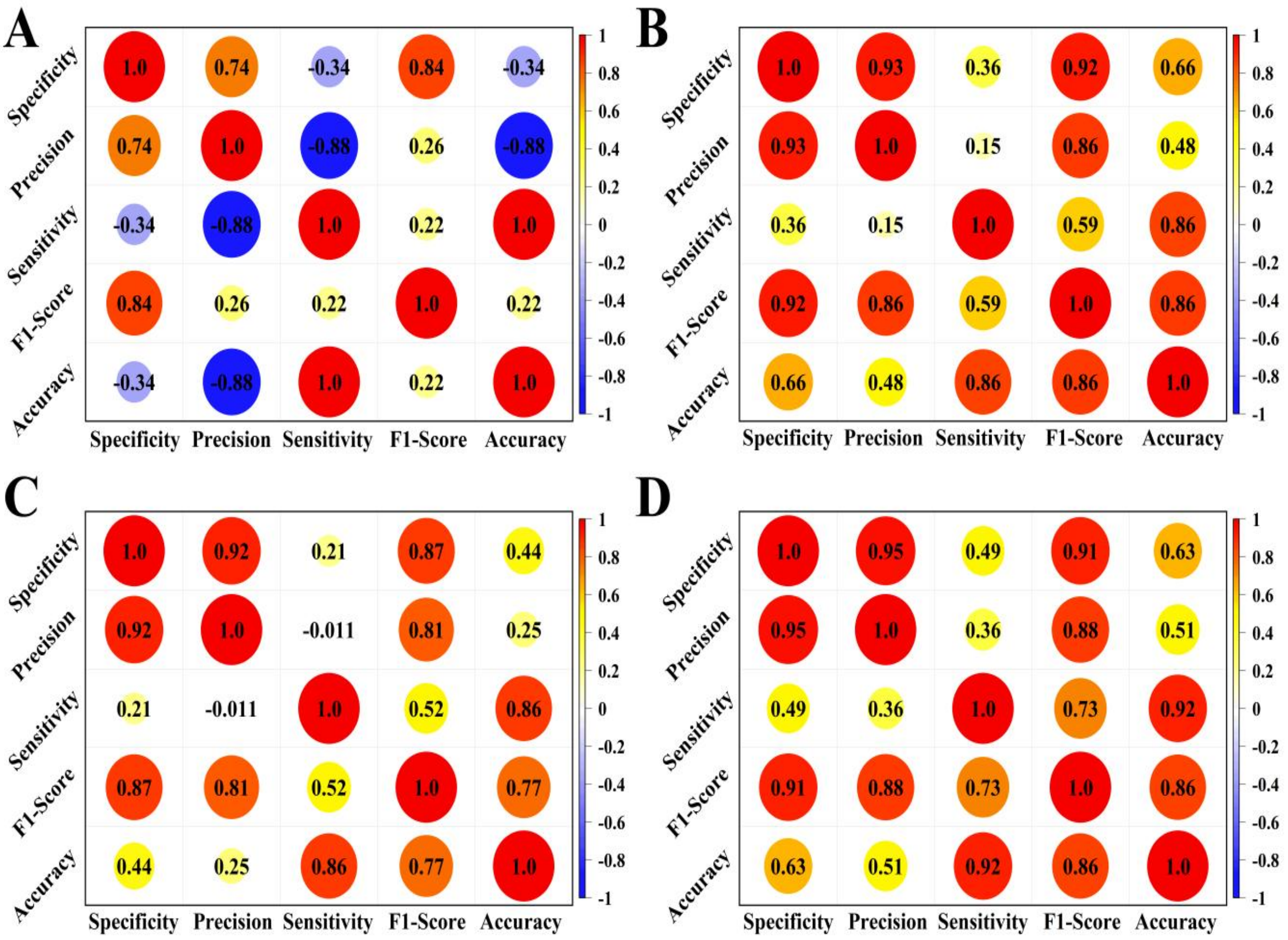

Appendix Fig. 1 Heatmaps of correlation among evaluation metrics of different models. A, B, C, and D represent the heatmap analysis results of five evaluation metrics for Improved ViT, Improved ViT+KAN, Improved ViT+BiFormer, and Improved ViT+KAN+BiFormer, respectively.

Appendix Fig. 1 shows ImprovedViT+KAN+BiFormer achieves the strongest positive correlations among five evaluation metrics, reflecting optimal inter-metric synergy. Compared to Improved ViT, it increases Specificity-Precision correlation by 0.21 and Sensitivity-F1-score correlation by 0.51. Relative to Improved ViT+KAN, it raises Sensitivity-Accuracy correlation by 0.06. These enhanced correlations demonstrate that combining KAN with BiFormer improves both feature extraction consistency and classification alignment.

Interval plots statistically visualize data trends and variability, facilitating multi-distribution comparison and statistical inference. Appendix Fig. 2 employs this method to display soybean disease recognition accuracy, with bars and error bands representing mean values and 95% confidence intervals respectively.

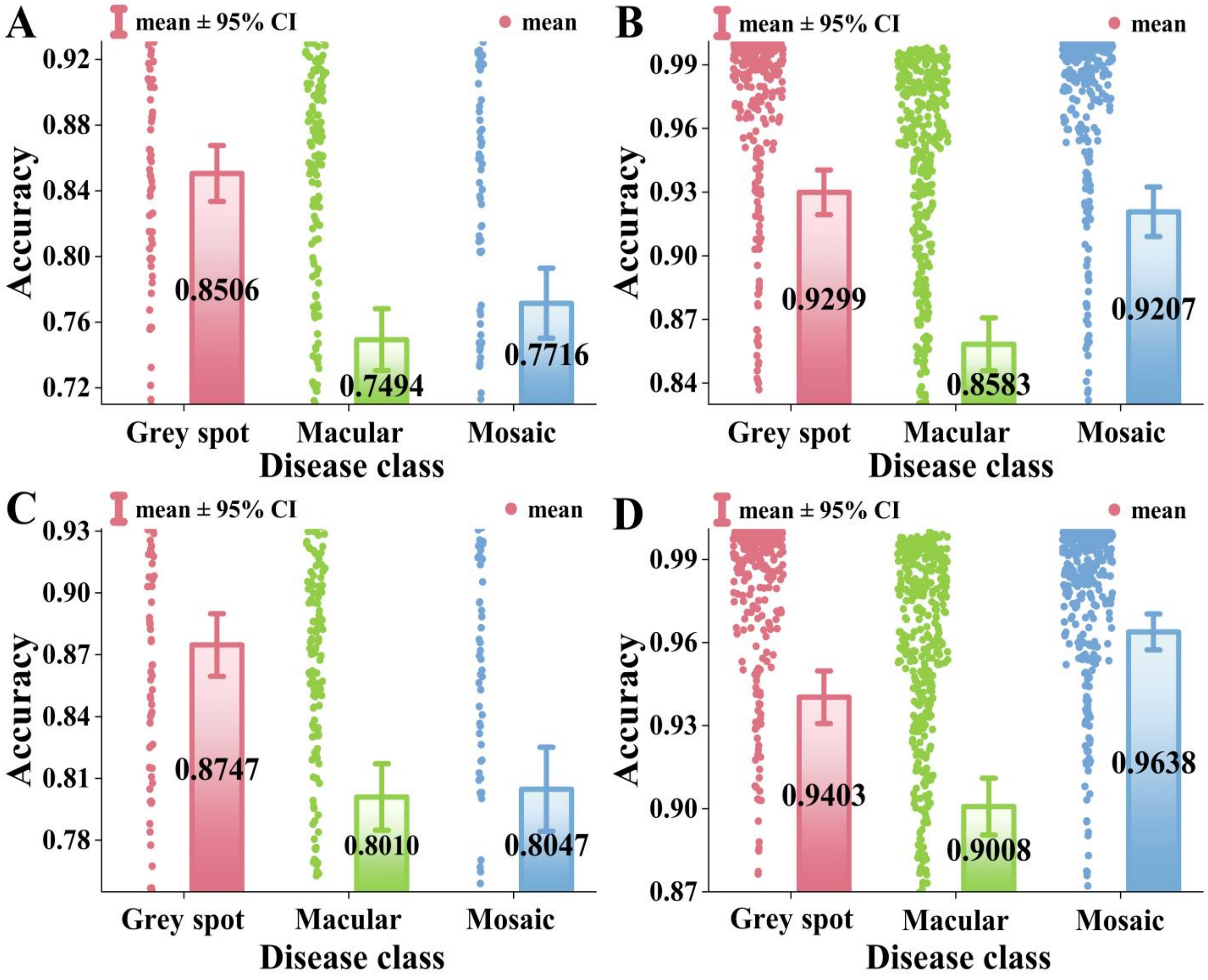

Appendix Fig. 2 displays mean confidence levels of four models: Improved ViT (A, ≥74.4%), Improved ViT+KAN (B, ≥85.2%), Improved ViT+BiFormer (C, >80.0%), and Improved ViT+KAN+BiFormer (D, ≥90.0%) across three disease categories.

Appendix Fig. 2 demonstrates that the proposed model achieves the most consistent confidence distributions across all three disease recognition tasks. It improves mean confidence by 15.6% over the lowest-performing baseline (Improved ViT) and by 4.8% over the strongest comparator (Improved ViT+KAN). While Improved ViT+BiFormer and Improved ViT+KAN show competitive performance in certain categories, their wider confidence intervals indicate lower prediction stability. The proposed model achieves superior performance, which can be attributed to the synergistic integration of KAN and BiFormer. This combination enhances the extraction of subtle lesion features, thereby improving prediction stability. These results confirm our model's superior confidence levels and enhanced prediction consistency, ensuring reliable performance for real-world agricultural applications.

To evaluate the proposed model for soybean leaf disease classification, we used Sensitivity, Precision, F1-score, and Specificity as key metrics and conducted systematic comparisons with

established architectures in Appendix Fig. 3.

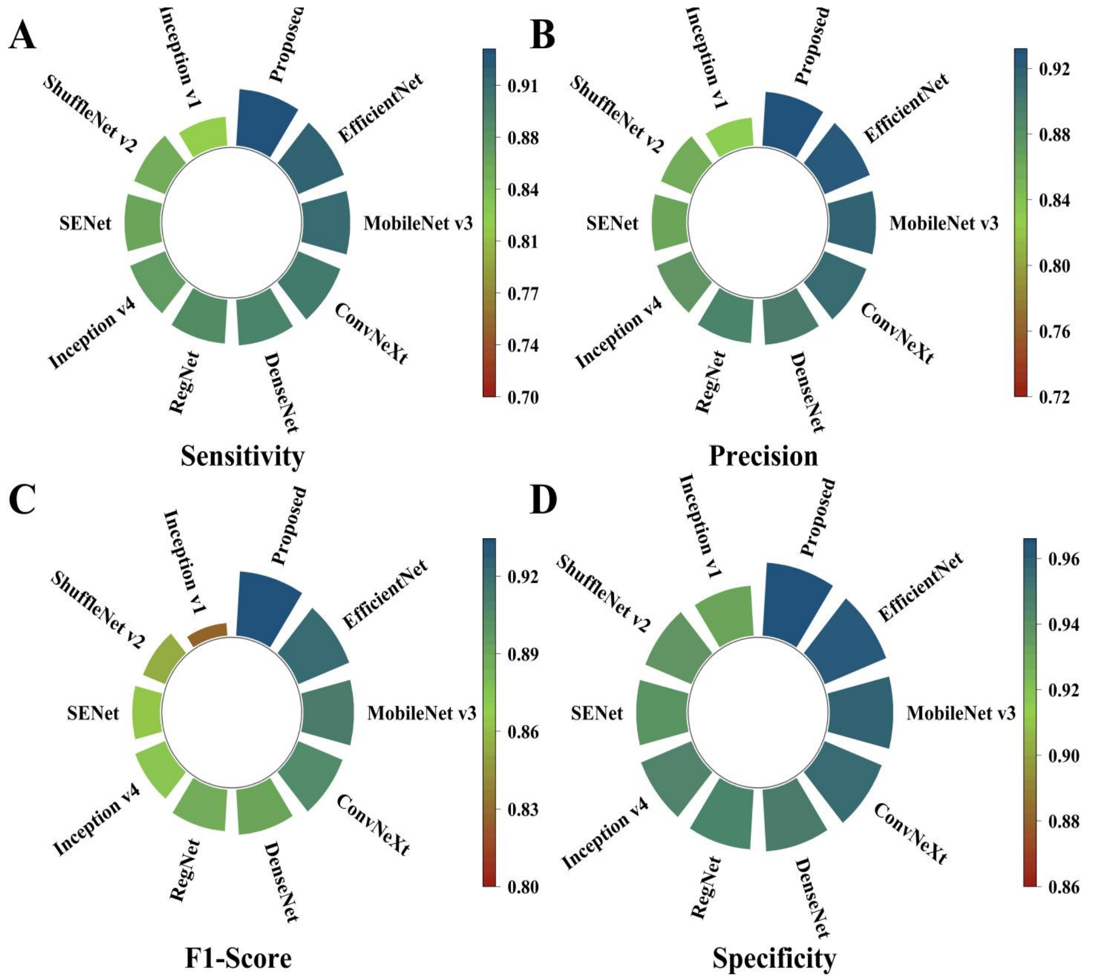

Appendix Fig. 3 compares performance metrics of established models. A is Sensitivity, B is Precision, C is F1-Score, and D is Specificity.

Appendix Fig. 3 compares model performance using radial bar charts (A – D). The proposed model demonstrates superior sensitivity relative to ShuffleNet v2, higher precision than Inception v1, and improved specificity compared to several modern architectures. It also achieves a longer radial bar in F1-score than RegNet, ConvNeXt, and EfficientNet. This performance advantage is attributed to the KAN module's ability to model subtle lesion features and the BiFormer module's capacity to precisely focus on symptom regions. As a result, the model performs consistently and excellently across all evaluation metrics, confirming its comprehensive superiority in soybean disease classification and its reliability for practical agricultural applications.
